# Pharmacokinetic Evaluation of New Drugs Using a Multi-Labelling Approach and PET Imaging: Application to a Drug Candidate with Potential Application in Neuromuscular Disorders

**DOI:** 10.3390/biomedicines11020253

**Published:** 2023-01-18

**Authors:** Rossana Passannante, Vanessa Gómez-Vallejo, Maialen Sagartzazu-Aizpurua, Laura Vignau Arsuaga, Pablo Marco-Moreno, Garazi Aldanondo, Ainara Vallejo-Illarramendi, Pablo Aguiar, Unai Cossío, Abraham Martín, Jonas Bergare, Lee Kingston, Charles S. Elmore, Miguel Angel Morcillo, Pablo Ferrón, Jesus M. Aizpurua, Jordi Llop

**Affiliations:** 1 CIC biomaGUNE, Basque Research and Technology Alliance (BRTA), 20014 San Sebastián, Spain; 2 Departamento de Química Orgánica-I, UPV/EHU-University of the Basque Country, 20018 San Sebastián, Spain; 3 Group of Neuromuscular Diseases, Biodonostia Health Research Institute, 20014 San Sebastián, Spain; 4 Deusto Physical TherapIker, Physical Therapy Department, Faculty of Health Sciences, University of Deusto, 20012 San Sebastián, Spain; 5 Group of Neuroscience, Department of Pediatrics, Hospital Donostia, UPV/EHU, 20014 San Sebastián, Spain; 6 Molecular Imaging Group, IDIS, CIMUS, Universidad de Santiago de Compostela, 15782 Santiago de Compostela, Spain; 7 Ikerbasque, Basque Foundation for Science, Maria Diaz de Haro 3, 48013 Bilbao, Spain; 8 Laboratory of Neuroimaging and Biomarkers of Inflammation, Achucarro Basque Center for Neuroscience, Science Park UPV/EHU, Sede Building B, Sarriena, 48940 Leioa, Spain; 9 Early Chemical Development, Pharmaceutical Sciences R&D, AstraZeneca, 431 83 Gothenburg, Sweden; 10 CIEMAT, Medical Applications of Ionizing Radiations Unit, 28040 Madrid, Spain; 11 Miramoon Pharma S.L., Avda Tolosa-72, 20018 San Sebastián, Spain

**Keywords:** PET, radiolabelling, pharmacokinetics

## Abstract

Background and objective: The determination of pharmacokinetic properties of new chemical entities is a key step in the process of drug development. Positron emission tomography (PET) is an ideal technique to obtain both biodistribution and pharmacokinetic parameters of new compounds over a wide range of chemical modalities. Here, we use a multi-radionuclide/multi-position labelling approach to investigate distribution, elimination, and metabolism of a triazole-based FKBP12 ligand (AHK2) with potential application in neuromuscular disorders. Methods: Target engagement and stabilizing capacity of the drug candidate (AHK2) towards FKBP12-RyR was evaluated using competitive ligand binding and proximity ligation assays, respectively. Subsequently, AHK2 was labelled either with the positron emitter carbon-11 (^11^C) via ^11^C-methylation to yield both [^11^C]AHK2.1 and [^11^C]AHK2.2, or by palladium-catalysed reduction of the corresponding 5-iodotriazole derivative using ^3^H gas to yield [^3^H]AHK2. Metabolism was first investigated in vitro using liver microsomes. PET imaging studies in rats after intravenous (IV) administration at different doses (1 µg/Kg and 5 mg/Kg) were combined with determination of arterial blood time-activity curves (TACs) and analysis of plasma samples by high performance liquid chromatography (HPLC) to quantify radioactive metabolites. Arterial TACs were obtained in continuous mode by using an in-house developed system that enables extracorporeal blood circulation and continuous measurement of radioactivity in the blood. Pharmacokinetic parameters were determined by non-compartmental modelling of the TACs. Results: In vitro studies indicate that AHK2 binds to FKBP12 at the rapamycin-binding pocket, presenting activity as a FKBP12/RyR stabilizer. [^11^C]AHK2.1, [^11^C]AHK2.2 and [^3^H]AHK2 could be obtained in overall non-decay corrected radiochemical yields of 14 ± 2%, 15 ± 2% and 0.05%, respectively. Molar activities were 60–110 GBq/µmol, 68–122 GBq/µmol and 0.4–0.5 GBq/μmol, respectively. In vitro results showed that oxidation of the thioether group into sulfoxide, demethylation of the CH_3_O-Ar residue and demethylation of –N(CH_3_)_2_ were the main metabolic pathways. Fast metabolism was observed in vivo. Pharmacokinetic parameters obtained from metabolite-corrected arterial blood TACs showed a short half-life (12.6 ± 3.3 min). Dynamic PET imaging showed elimination via urine when [^11^C]AHK2.2 was administered, probably reflecting the biodistribution of [^11^C]methanol as the major metabolite. Contrarily, accumulation in the gastrointestinal track was observed after administration of [^11^C]AKH2.1. Conclusions: AHK2 binds to FKBP12 at the rapamycin-binding pocket, presenting activity as a FKBP12/RyR stabilizer. Studies performed with the ^3^H- and ^11^C-labelled FKBP12/RyR stabilizer AHK2 confirm fast blood clearance, linear pharmacokinetics and rapid metabolism involving oxidation of the sulfide and amine moieties and oxidative demethylation of the CH_3_-O-Ar and tertiary amine groups as the main pathways. PET studies suggest that knowledge about metabolic pathways is paramount to interpret images.

## 1. Introduction

Appropriate pharmacokinetic profiling is a critical step in the drug development process. In preclinical phases, pharmacokinetic parameters can be first predicted using in silico models and then determined using both in vitro and in vivo models [1]. In silico models predict drug behaviour based on physicochemical properties of drug candidates in combination with crystal structures of a protein (target) and a database of ADME (absorption, distribution, metabolism, and elimination) properties generated in laboratories [2]. These studies are often capable of faithfully predicting absorption, target organ concentration, clearance, efficacy, and toxicity or other adverse effects early in the drug discovery and development processes [3,4]. In vitro pharmacokinetics assays include permeability and metabolic stability. Permeability can be characterized by using cells and membrane vesicles from organs that usually express a high concentration of transporters. The use of the human colon cancer derived Caco-2 cell line [5,6] and Madin Darby canine kidney cells with the MDR1 gene (MDCK-MDR1), the gene encoding for the efflux protein, P-glycoprotein (P-gp) [7,8], is widely established to predict in vivo absorption. Metabolic stability generally comprises studies using primary hepatocyte cultures or sub-cellular fractions isolated from them [9]. 

Despite the value of both in silico and in vitro models, in vivo studies in different animal species are required before entering clinical phases. Pharmacokinetic studies in animals usually involve the use of high-performance liquid chromatography combined with mass spectrometry (HPLC-MS) as the analytical technique [10]. After the administration of the drug to the animal using the designed administration route, body fluids (i.e., blood, urine) or organs (these after animal sacrifice) can be extracted and, after appropriate processing, the resulting samples are analysed to obtain information about the concentration of the test compound and/or metabolites in order to gather pharmacokinetic information. This approach provides accurate and exhaustive information. However, the main drawbacks include invasiveness and the need for a large number of animals, with the consequent economic and ethical burden.

One approach that is gaining relevance in the drug development process is the use of nuclear imaging techniques, particularly positron emission tomography (PET), to assess biodistribution and extract pharmacokinetic information [11,12]. PET is an ultrasensitive and minimally invasive technique, which relies on the administration of a chemical or biological entity labelled with a positron emitter to a living organism. The emission of gamma rays resulting from positron annihilation allow for the generation of time-resolved three-dimensional images, which provide information about the concentration of the labelled compound at the whole-body level. Complementary to PET imaging studies, body fluid samples can be withdrawn at pre-defined time points to obtain quantitative data about the concentration of parent compound and radiolabelled metabolites [13].

Ryanodine receptors type 1 (RyR1) are homotetrameric channels at the sarco/endoplasmic reticulum that act as calcium release channels and participate in muscle contraction. FK506 binding protein 12 (FKBP12) is a ubiquitous immunophilin that interacts with both FK506 and Rapamycin, which inhibit its prolyl isomerase activity. The FKBP12-FK506 complex binds calcineurin, while the FKBP12-Rapamycin complex binds to the mammalian target of Rapamycin (mTOR) at the FRB domain [14]. Most interestingly, FKBP12 is one of the most studied RyR1 modulators and is known to coordinate cooperative gating of RyR1 subunits, eliminating subconductance states and stabilizing the fully open and closed state of the channel. Mutations or post-translational modifications affecting FKBP12/RyR1 interaction may alter calcium homeostasis by inducing calcium leakage from the RyR1 channels into the cytosol. This pathogenic mechanism underlies several neuromuscular conditions including muscular dystrophies, RyR1-related myopathies, or sarcopenia [15,16,17,18]. We work under the hypothesis that FKBP12 ligands may alleviate this cascade of pathological events and slow disease progression, by rescuing FKBP12/RyR1 interaction and normalizing calcium homeostasis [19].

In this work, we report on the combination of PET imaging with complementary radio-analytical techniques to investigate pharmacokinetic properties of a novel triazole-based FKBP12 ligand, namely, AHK2, with potential application for the treatment of neuromuscular disorders, such as Duchenne muscular dystrophy, a lethal genetic muscular disease.

## 2. Materials and Methods 

### 2.1. Chemistry

Sodium azide, 2-chloroethyl-N,N-dimethylammonium chloride, 2-chloroethyl-N-methylammonium chloride, copper (I) acetate, copper (I) iodide, sodium acetate, sodium ascorbate, sodium carbonate, potassium carbonate, thiophenol, N-methylpyrrolidone, N-bromosuccinimide and N,N-diisopropylethylamine were purchased from Alfa Aesar (Haverhill, MA, USA) and Merk KGaA (Darmstadt, Germany) and were used without further purification. Reagent grade methanol and anhydrous acetonitrile were purchased from Scharlab (Barcelona, Spain). 4-Methoxyphenyl propargyl sulfide was prepared as previously described (Heterocycles 1994, 39, 371). Flash chromatography was performed using Merck silica gel 60 (230–400 mesh). ^1^H NMR and ^13^C NMR spectra were recorded at 20 ºC on Bruker Avance spectrometers operated at 500 MHz and 400 MHz for ^1^H and at 125 MHz and 101 MHz for ^13^C, respectively. All ^1^H NMR spectra were reported in parts per million (ppm) downfield of TMS or were measured relative to the residual signals for CHCl_3_ (7.26 ppm for ^1^H and 77.0 ppm for ^13^C). Melting points were measured using open glass capillaries in a Büchi SMP-20 apparatus. Mass spectra were acquired on a time of flight (TOF) mass spectrometer (SYNAPT G2 HDMS from Waters) equipped with an electrospray source in positive mode (ESI+). Infrared spectra were recorded on a Bruker Alpha P spectrometer.

Compound AHK2: 1-[2-(*N*,*N*-dimethylamino)ethyl]-4-[(4-methoxyphenyl)thiomethyl]-1H-1,2,3-triazole. 

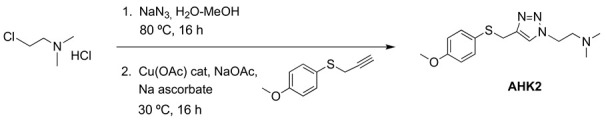



A solution of 2-chloroethyl-*N*,*N*-dimethylammonium chloride (36 mmol, 5.18 g) and sodium azide (39 mmol, 2.52 g) in water (30 mL) was heated at 80 °C overnight. To the resulting solution of 2-azidoethyl-*N*,*N*-dimethylamine hydrochloride was successively added 4-methoxyphenyl propargyl sulfide (30 mmol, 5.35 g) dissolved in MeOH (120 mL), water (25 mL), CuOAc (1.5 mmol, 180 mg), NaOAc (90 mmol, 7.38 g) and sodium ascorbate (15 mmol, 2.97 g), and the mixture was stirred at 30 °C overnight. Organic solvents were evaporated, and the remaining aqueous solution was stirred with 20% ammonia (50 mL) for 30 min and was extracted with EtOAc (3 × 70 mL). The organic phase was acidified with 2M HCl and the aqueous layer was washed with EtOAc (80 mL). The aqueous phase was basified with 20% Na_2_CO_3_ and extracted with EtOAc (3 × 60 mL). The combined organic extract was dried (Na_2_SO_4_) and evaporated at reduced pressure. The crude product was purified by column chromatography (silica gel, CH_2_Cl_2_/MeOH 95:5). Yield 7.53 g (86%). White solid (Mp: 35–40 °C). ^1^H NMR (400 MHz, CD_3_OD) δ 7.67 (s, 1H), 7.29 (d, *J* = 8.7 Hz, 2H), 6.85 (d, *J* = 6.9 Hz, 2H), 4.44 (t, *J* = 6.5 Hz, 2H), 4.09 (s, 2H), 3.77 (s, 3H), 2.75 (t, *J* = 6.5 Hz, 2H), 2.27 (s, 6H). ^13^C NMR (101 MHz, CD_3_OD): 159.2, 144.8, 133.7, 125.5, 122.6, 114.5, 58.6, 55.3, 48.0, 45.3, 30.9. HRMS: calculated for (M + H) C_14_H_21_N_4_OS 293.1436 found: 293.1440.

Precursor 1: 1-[2-(N-methylamino)ethyl]-4-[(4-methoxyphenyl)thiomethyl]-1H-1,2,3-triazole. 

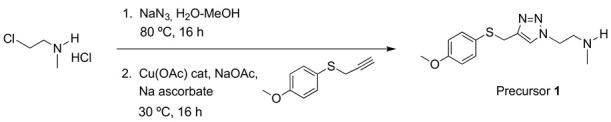



The same procedure to prepare AHK2 was followed starting from 2-chloroethyl-*N*-methylammonium chloride (4.8 mmol, 624 mg), 4-methoxyphenyl propargyl sulfide (4.0 mmol, 713 mg), CuOAc (0.2 mmol, 24 mg), NaOAc (12 mmol, 981 mg) and sodium ascorbate (2 mmol, 395 mg). The resulting crude was purified by column chromatography (CH_2_Cl_2_/MeOH 95:5 then 90:10). Yield: 1.00 g (90 %). Yellowish oil. ^1^H NMR (400 MHz, CD_3_OD) δ 7.65 (s, 1H), 7.30 (d, *J* = 8.8 Hz, 2H), 6.87 (d, *J* = 8.8 Hz, 2H), 4.45 (t, *J* = 6.2 Hz, 2H), 4.10 (s, 2H), 3.79 (s, 3H), 2.99 (t, *J* = 6.2 Hz, 2H), 2.38 (s, 3H). ^13^C NMR (101 MHz, CD_3_OD) δ 161.0, 146.1, 135.3, 126.4, 124.7, 115.6, 55.8, 51.6, 50.4, 35.7, 31.2. IR (cm^−1^): 2940, 1591, 1493, 1284, 1241, 1026, 824. HRMS: Calculated for C_13_H_18_N_4_OS 278.1201 found: 278.1191.

Precursor 2: 1-[2-(*N*,*N*-dimethylamino)ethyl]-4-[(4-hydroxyphenyl)thiomethyl]-1H-1,2,3-triazole. 

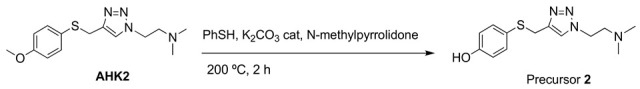



A solution of AHK2 (1.26 mmol, 370 mg), thiophenol (3 mmol, 0.25 mL) and potassium carbonate (0.05 mmol, 7 mg) in N-methylpyrrolidone (2.5 mL) was heated at 200 °C for 2 h in an ACE pressure tube under inert atmosphere. The solvent was evaporated under high vacuum and the product was purified by column chromatography (CH_2_Cl_2_/MeOH 95:5). Yield. (57%). White solid (Mp: 102 °C). ^1^H NMR (400 MHz, CDCl_3_) δ 7.49 (s, 1H), 7.16 (d, *J* = 8.5 Hz, 2H), 6.67 (d, *J* = 8.5 Hz, 2H), 4.47–4.37 (m, 2H), 4.05 (s, 2H), 2.76 (t, *J* = 6.4 Hz, 2H), 2.28 (s, 6H). ^13^C NMR (101 MHz, CDCl_3_) δ 157.1, 145.2, 134.5, 122.9, 122.9, 116.3, 58.4, 48.1, 45.2, 30.7. HRMS: Calculated for C_13_H_18_N_4_OS 278.1201 found: 278.12010.

Precursor 3: 1-[2-(*N*,*N*-dimethylamino)ethyl]-5-iodo-4-[(4-methoxyphenyl) thiomethyl] -1H-1,2,3-triazole. 

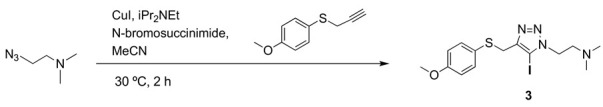



To a solution of 4-methoxyphenyl propargyl sulfide (3.84 mmol, 648 mg), CuI (4.3 mmol, 831 mg), NBS (4.6 mmol, 4.6 mg), DIPEA (4.3 mmol, 756 µL) in dry CH_3_CN (10 mL) kept under nitrogen atmosphere, was added 2-azido-*N*,*N*-dimethylethyl-1-amine (4.2 mmol, 482 mg), and the reaction mixture was stirred at room temperature for 2 h. Then, solvent was evaporated under reduced pressure, the residue was suspended in aqueous saturated NaCl (15 mL) and the aqueous suspension was extracted with CH_2_Cl_2_ (3 × 15 mL). The combined organic layers were washed with aqueous saturated NaCl (15 mL), dried over MgSO_4_ and concentrated under reduced pressure. The product was purified by column chromatography. (CH_2_Cl_2_/MeOH 20:1). Yield 0.92 g (62%). Brown solid (Mp: 45 °C). ^1^H NMR (400 MHz, CD_3_OD) δ 7.26 (d, 2H), 6.84 (d, 2H), 4.50 (t, *J* = 6.9 Hz, 2H), 4.01 (s, 2H), 3.79 (s, 3H), 2.80 (t, *J* = 6.9 Hz, 2H), 2.32 (s, 6H). ^13^C NMR (101 MHz, CD_3_OD) δ 159.3, 148.0, 134.7, 124.4, 114.1, 79.6, 57.9, 54.9, 48.2, 45.1, 31.2. IR (cm^−1^): 2946, 2830, 2779, 1709, 1589, 1491, 1461, 1439, 1283, 1241, 1173, 1121, 1101, 1021, 858, 821, 788, 640, 522, 449. HRMS: Calculated for C_14_H_19_IN_4_OS 418.0324; found: 418.0325.

### 2.2. Biological Evaluation

#### 2.2.1. Competitive Ligand Binding Assay of AHK2

Target engagement of AHK2 to FKBP12 was demonstrated by a competitive ligation assay as previously described [19]. Briefly, HEK293 (ATCC) cells were transfected with FKBP/FRB NanoBiT Control Pair (Promega, Madison, WI, USA) and kinetic measurements were performed 24 h after transfection with Nano-Glo Live Cell Reagent (Promega), as described by the manufacturer. A total of 30 nM rapamycin (Sigma-Aldrich, Madrid, Spain) and AHK2 (1 μM–10 mM) was sequentially added and each effect was recorded for 10 min. Specific activity was normalized to vehicle, and IC50 was calculated using GraphPad Prism by fitting the data to a four-parameter logistic curve.

#### 2.2.2. In Situ Proximity Ligation Assay (PLA)

In situ PLA was used for evaluation of FKBP12/RyR stability in human myotubes under nitro-oxidative stress, as previously described [19]. Briefly, immortalized human myoblasts (Myology Institute, Paris, France) were differentiated into myotubes and nitro-oxidative stress was induced by addition of the peroxynitrite donor 3-morpholinosydnonimine (SIN1, 5 mM, SCBT, Heidelberg, Germany) for 30 min. Myotubes were fixed with 4% paraformaldehyde (Aname, Madrid, Spain) and incubated overnight with the following primary antibodies: anti-RyR mouse mAb (1:200, Thermo Scientific, Waltham, MA, USA), and anti-FKBP12 rabbit pAb (1:100, Novus Biologicals, Centennial, CO, USA). The Duolink in situ PLA Orange assay kit was used with corresponding conjugated secondary antibodies (Sigma). Samples were counterstained with FITC-conjugated Myosin Heavy Chain-CFS mAb (MyHC, 1:50, R&D, Minneapolis, MN, USA) and mounted with ProLong Gold antifade reagent (Life Technologies, Eugene, OR, USA). Image quantification was performed with ImageJ (NIH). For each image, the total number of spots was normalized to the MyHC area. At least 3 images per condition were analysed with an average of 8–9 myotubes per image. Statistical significance was determined using One-Way ANOVA followed by Dunett’s multiple comparisons test. *p*-values < 0.05 were considered statistically significant.

### 2.3. Radiochemistry

#### 2.3.1. Radiolabelling with carbon-11

**Scheme 1:**
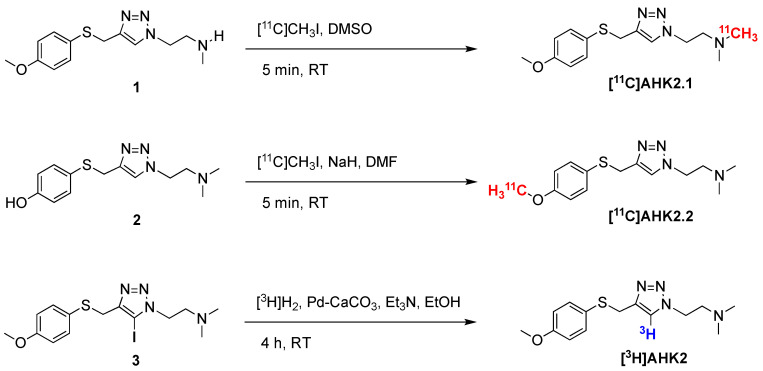
Radiosynthetic process for the preparation of [^11^C]AHK2.1, [^11^C]AHK2.2 and [^3^H]AHK2; RT: room temperature.

Radiolabelling of AHK2 with carbon-11 (^11^C) was carried out via ^11^C-methylation in two different positions using [^11^C]CH_3_I as the labelling agent (Scheme 1). The synthesis of [^11^C]CH_3_I was carried out, in all cases, using a TRACERlab FX_C Pro_ synthesis module (GE Healthcare, Waukesha, WI, USA). [^11^C]CH_4_ was generated in an IBA Cyclone 18/9 cyclotron by proton irradiation (target current = 22 µA, integrated current = 2 µAh) of a N_2_/H_2_ gas mixture (99/1, starting pressure = 16 bar) with high energy (18 MeV) protons. The radioactive gas was trapped in Carbosphere 60/80 (Alltech Associates, Inc., Vienna, Virginia, US) at T = −140 °C, desorbed by heating (80 °C) and allowed to react with iodine at 720 °C to form [^11^C]CH_3_I in a gas circulating process. [^11^C]CH_3_I was selectively retained in a trap containing Porapak^TM^ Q (50–80 mesh, Waters Corporation) at room temperature, while unreacted [^11^C]CH_4_ was recirculated. At the end of the process (8–9 cycles), the Porapak^TM^ Q trap was heated at 190 °C and [^11^C]CH_3_I was distilled under continuous helium flow (20 mL/min, 2.5 min). The gas stream was passed through a trap containing phosphorous pentoxide and Ascarite II^®^ (20–30 mesh) before being introduced in a 2 mL stainless steel loop, prefilled with a solution of the appropriate precursor.

For the preparation of [^11^C]AHK2.1 (see Scheme 1), the loop was filled with precursor 1 (1 mg) in 80 μL dimethylsulfoxide. After trapping of the [^11^C]CH_3_I, the reaction was allowed to occur for 5 min at room temperature. The reaction mixture was purified by high performance liquid chromatography (HPLC), using a Phenomenex Luna C18 column (250 × 10mm, 5 µm) as the stationary phase and 10 mM ammonium formate solution (AMF; pH = 8.3)/acetonitrile (50/50) as the mobile phase (flow rate = 5 mL/min, retention time = 6.5 min; see Figure S1c for representative chromatographic profile). For the preparation of AHK2.2, the reaction loop was prefilled with a solution of precursor 2 (2 mg) in 100 μL anhydrous dimethylformamide (DMF) containing 1.7 mg of NaH (60% dispersion in mineral oil). After trapping of the [^11^C]CH_3_I, the reaction was allowed to occur for 5 min at room temperature. The reaction mixture was purified by HPLC, under the same conditions as above (see Figure S2c for representative chromatographic profile).

In both cases, the desired fraction (monitored by using a radioactivity detector) was collected, diluted with ultrapure water (20 mL, obtained from a Milli-Q^®^ Purification System, Millipore^®^, Merck KGaA, Darmstadt, Germany) and flushed through a C-18 cartridge (Sep-Pak^®^ Light, Waters, Milford, MA, USA) to selectively retain the labelled compound. The cartridge was washed with ultrapure water (5 mL) and the labelled compound was finally eluted with ethanol (1 mL) and reconstituted with physiologic solution (9 mL). The amount of radioactivity of the final radiotracer was measured in a dose calibrator (PETDOSE HC, Comecer) and a sample was submitted to quality control.

The radiochemical purity and the molar activity were determined by HPLC, using an Agilent 1200 Series HPLC system with a multiple wavelength UV detector (λ = 254 nm) and a radiometric detector (Gabi, Raytest). A RP-C18 column (Mediterranean Sea18, 4.6 × 150 mm, 5 μm particle size) was used as the stationary phase and 10 mM AMF solution (pH = 8.3)/acetonitrile (50/50, flow rate 1 mL/min) was used as the mobile phase (retention time = 4.2 min). The same method was used to monitor radiochemical conversion in synthesis performed during optimisation of experimental conditions (see Figures S1b and S2b for representative chromatographic profiles corresponding to compounds [^11^C]AHK2.1 and [^11^C]AHK2.2, respectively).

#### 2.3.2. Radiolabelling with hydrogen-3

Radiolabelling with hydrogen-3 (^3^H) was achieved by palladium-catalysed hydrogenation of the 5-iodotriazole precursor (Scheme 1). Precursor 3 (1 mg. 2.31 µmol), 10% palladium on calcium carbonate (1 mg, Sigma-Aldrich, St. Louis, MO, USA) and triethylamine (10 μL, Sigma-Aldrich, St. Louis, MO, USA) in ethanol (1 mL, absolute, 99.5%) were degassed by three freeze-pump-thaw cycles. The flask was filled with tritium gas (448 GBq, 0.2 mmol), released from the uranium bed by heating it with a blowtorch, and stirred at room temperature for 4 h. After this time, the flask was frozen, and the excess of tritium gas was recovered on a uranium recovery bed. The flask was warmed to room temperature and the solvent and residual tritium gas was removed under a nitrogen flow. The reaction mixture was taken up in methanol and then passed through a syringe filter (0.45 μm PTFE filter). The solvent was evaporated under reduced pressure to yield a solid residue, which was purified via preparative HPLC using an Xbridge Prep C-18 column (5 μm OBD 19 × 100 mm) as the stationary phase and AMF solution (pH = 10)/acetonitrile as the mobile phase. Chromatographic runs were performed under gradient conditions (75:25 AMF:acetonitrile → 25:75 AMF:acetonitrile) with a total chromatographic time of 30 min (flow rate = 10 mL/min). The purified fractions were dried under vacuum and reconstituted in 10 mL of ethanol and stored at −80 °C. The amount of radioactivity of the final radiotracer was measured in a scintillation counter (BECKMAN LS6500, scintillation fluid ULTIMA GOLD) and a sample was submitted to quality control. The radiochemical purity was determined to be >99.5% by HPLC. A CSH C18 column (Waters XSelect 3 × 100 mm, 2.5 μm particle size) was used as the stationary phase and AMF solution (pH = 10)/acetonitrile was used as the mobile phase under gradient conditions (0–3 min: 5% acetonitrile; 3–20 min: from 5% to 95% acetonitrile; 25–30 min: 95% acetonitrile), followed by a 5-minute wash with 100% acetonitrile at a flow rate of 1 mL/min (retention time = 13.2 min). The identity of the ^3^H-labelled compound was confirmed by liquid chromatography-mass spectrometry (LC-MS) and ^3^H-NMR. For the LC-MS analysis, a CSH C18 column (Waters XSelect 4.6 × 150 mm, 3.5 μm particle size) was used as the stationary phase and AMF solution (pH = 10)/acetonitrile was used as the mobile phase under gradient conditions (0–3 min: 5% acetonitrile; 3–8 min: 5 to 95% acetonitrile; 8–10 min: 95% acetonitrile), followed by a 2 min wash with 100% acetonitrile at a flow rate of 1 mL/min (retention time = 3.6 min). ^3^H NMR (533 MHz, MeOD): δ = 7.78 (s, 1 ^3^H).

### 2.4. In Vitro Studies

#### In Vitro Metabolism

Labelled compounds ([^11^C]AHK2.1, 2.8 MBq, 0.4 nmol; [^11^C]AHK2.2, 4.7–5.1 MBq, 0.2–0.4 nmol; [^3^H]AHK2, 0.25 MBq, 0.5 nmol; or non-radioactive AHK2, the latter for metabolite identification) and microsomal protein (rat Sprague Dawley microsomes, Thermo Fisher Scientific, Carlsbad, CA, USA; which are a sub-cellular fraction of hepatocytes containing enzymes responsible for the metabolism of over 90% of commercial drugs; 20 mg/mL) were pre-incubated for 5 min with gentle agitation (300 rpm) in 100 mM phosphate buffer (pH 7.4). The reaction was initiated by the addition of 10 μL freshly prepared 20 mM nicotinamide adenine dinucleotide phosphate (NADPH, Roche, Mannheim, Germany) in 100 mM phosphate buffer (pH 7.4). At t = 30 min, the reaction was terminated by the addition of 200 μL of acetonitrile. Samples were centrifuged at 5800× *g* rpm for 5 min to pellet the precipitated protein. The resulting supernatants were analysed by HPLC, using an Agilent 1200 Series chromatograph equipped with a multiple wavelength UV detector (λ = 254 nm) and two radioactivity detectors, one of them equipped with a solid scintillation cell, especially designed to detect low energy β^-^ (Ramona, Raytest), and one of them equipped with a NaI crystal, for the detection of γ-rays (Gabi, Raytest). A RP-C18 column (Mediterranean Sea18, 4.6 × 150 mm, 5 μm particle size) was used as the stationary phase, with an Eclipse XDB-C18 (4.6 × 12.5 mm, 5 μm particle size) guard column. As the mobile phase, 10 mM AMF solution (pH = 8.3)/acetonitrile was used under gradient conditions (0–5 min: 5% acetonitrile; 5–30 min: from 5% to 50% acetonitrile; 30–35 min: from 50% to 5% acetonitrile) at a flow rate of 1 mL/min, with a total chromatographic time of 35 min. For the non-radioactive AHK2, the resulting supernatants were analysed by ultra-high-performance liquid chromatography coupled with electrospray ionization time-of-flight mass spectrometry (UHPLC-ESI-TOF-MS, Electrospray positive mode/W mode *m*/*z* range 100–1000). An Acquity C18 column (5 × 2.1 mm, 1.7µm particle size) was used as the stationary phase. As the mobile phase, 0.1% formic acid-H_2_O/acetonitrile was used under gradient conditions (0–0.5 min: 2% acetonitrile; 0.5–7.5 min: from 2% to 99% acetonitrile; from 7.5 to 10: from 99% to 2% acetonitrile) at a flow rate of 300 µL/min, with a total chromatographic time of 10 min.

### 2.5. In Vivo Studies

#### 2.5.1. General Considerations

Female Sprague-Dawley rats (8–9 weeks old, ca. 200 g, Janvier Labs, Saint-Berthevin, France) were used. The animals were maintained and handled in accordance with the Guidelines for Accommodation and Care of Animals (European Convention for the Protection of Vertebrate Animals Used for Experimental and Other Scientific Purposes) and internal guidelines. All experimental procedures were approved by the ethical committee of CIC biomaGUNE and local authorities before conducting experimental work. All animals were housed in ventilated cages and fed on a standard diet ad libitum.

#### 2.5.2. Extracorporeal Blood Circulation: Animal Surgery

**Figure 1:**
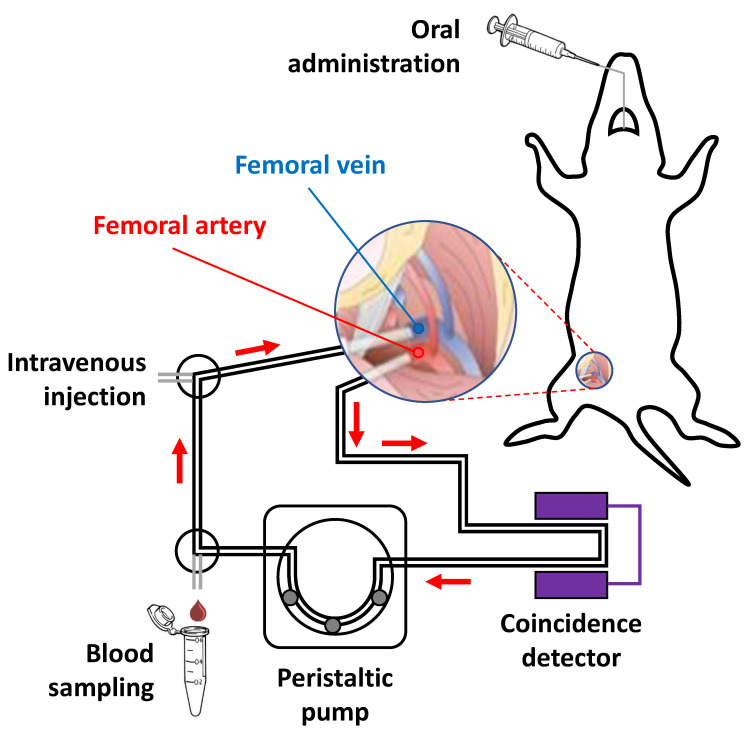
Schematic representation of experimental setting used for the extracorporeal blood circulation. Two catheters were first inserted into the femoral artery and vein, respectively. The blood was pumped through a radioactive (coincidence) detector using a peristaltic pump.

Anaesthesia was induced with 5% isoflurane (IsoFlo^®^, Abbott Laboratories, Abbott Park, IL, USA) and maintained by 2% of isoflurane in 100% O_2_. During the experiment, rats were kept normothermic using a heating blanket (Homeothermic Blanket Control Unit; Bruker). The rat was positioned in supine position on an operating table and the two distal hind limbs were fixed forming an approximately 30° angle with the horizontal plane. The surgical region was scrubbed using Betadine, starting in the centre, and making a circular sweep outward. A skin incision was made in the femoral region. Soft tissue was dissected to expose the femoral neurovascular bundle, consisting of femoral vein, artery, and nerve. Fine tip forceps were placed between the artery, vein and nerve and were slowly opened to separate the vessels, exposing an approximately 1 cm length section of artery/vein. The vena profunda femoris was sutured to avoid retrograde bleeding via this vessel, and the sapheno-femoral junction was cauterized. A few millimetres from venatomy, a vascular clip was inserted in the direction of the catheter (to control the flow) and a suture was applied in the opposite direction (to avoid retrograde bleeding). The same operation was carried out both on the femoral vein and femoral artery. An appropriate syringe filled with 50 U/mL Heparin/physiologic saline solution was fixed at the terminus of the catheters and these were filled with heparinized saline solution. On the upper half of the vessel circumference, at a 45° angle, an incision was made and the catheter (fine bore polythene tubing: ID 0.58 mm, OD 0.98 mm) was inserted through the venotomy and advanced to the vascular clip. The vascular clip was then removed, and the catheter advanced distally towards the level of the inguinal ligament. The catheter was finally secured to the femoral vein/artery proximally and distally with 2 single knots using 4–0 braided silk suture. The catheters were connected to an in-house developed system composed by a peristaltic pump and gamma-radiation coincidence detector (Bioscan, B-FC-4100). The femoral artery catheter, the gamma detector, the peristaltic pump, and the femoral vein catheter were thus connected in series, to enable the extracorporeal circulation of blood (flow 150 μL/s from femoral artery to femoral vein) and continuous measurement of radioactivity in the blood (Figure 1).

#### 2.5.3. Administration of Labelled Compounds, Blood Sampling and Determination of In Vivo Metabolism

The labelled compounds were administrated two different ways: (i) intravenous bolus infusion (ca. 37 MBq in 300 μL of saline solution was injected through the femoral vein catheter) and (ii) oral bolus administration. For oral administration, animals were fasted for 5 h. The radioactive dose (ca. 74 MBq in 300 μL of saline solution) was administered using flexible 20-gauge disposable flexible PTFE feeding needles (Fine Science Tools, F.S.T.) and a small animal Laryngoscope (Penn-Century, Model LS-2) for the correct visualization of the oesophagus. The study was conducted under no carrier added (NCA, dose adjusted to 1 μg/Kg) and carrier added (CA, dose adjusted to 5 mg/Kg) conditions. In all cases, data were imported in real time to an excel sheet using PLX-DAX add-on for Microsoft Excel (measured in volts per second).

Arterial blood samples (1 per animal) were withdrawn (see Figure 1) from the femoral artery catheter and introduced into a capillary (Microhaematocrit capillaries 75 × 1.55, Nahita). Samples were centrifuged at 11,000× *g* rpm for 10 min and the haematocrit was determined. A second blood sample was withdrawn and the amount of radioactivity of a fixed volume (10 µL) was measured in an automatic gamma counter (2470 Wizard, PerkinElmer) to determine *C_b_* (concentration of radioactivity in blood) and convert data imported with PLX-DAX (Volts per seconds) into Bq/s using a previously determined calibration curve. Values were then expressed as percentage of injected dose per gram of blood (% ID/g). The blood sample was then centrifuged at 14,800× *g* rpm for 10 min to separate the plasma, and the amount of radioactivity of a fixed volume (10 µL) was measured in an automatic gamma counter (2470 Wizard, PerkinElmer) to determine *C_p_* (concentration of radioactivity in plasma). The value *C_p_*⁄*C_b_* was used to transform the arterial blood TAC into arterial plasma TAC, as percentage of injected dose per gram of plasma (% ID/g plasma). This calculation assumes that the distribution of radioactivity between plasma and blood remains constant over time.

At different time points after administration of the labelled compounds, and simultaneously with the determination of the arterial blood TAC, arterial blood samples (150 µL) were withdrawn. Blood samples were processed to separate the plasma, which was further processed to determine the presence of radioactive metabolites. With that aim, plasma fractions were diluted with the same volume of acetonitrile. After mixing vigorously for 20 s, samples were centrifuged at 14,800× *g* rpm for 4 min. The liquid phase was separated from the precipitate by decantation and was analysed by HPLC, using an Agilent 1200 Series HPLC system with a multiple wavelength UV detector (λ = 254 nm) and a radiometric detector (Gabi, Raytest). An RP-C18 column (Mediterranean Sea18, 4.6 × 150 mm, 5 μm particle size) was used as the stationary phase, with an Eclipse XDB-C18 (4.6 × 12.5 mm, 5 μm particle size) guard column. The mobile phase was 10 mM AMF solution (pH = 8.3)/acetonitrile. In all cases, chromatographic runs were performed under gradient conditions, at a flow rate of 1 mL/min, with a total chromatographic time of 35 min (0–5 min: 5% acetonitrile; 5–30 min: from 5% to 50% acetonitrile; 30–35 min: from 50% to 5% acetonitrile). The percentage of parent compound present in plasma was calculated as the ratio between the area under the peak corresponding to AHK2 and the sum of the areas of all peaks in the chromatogram (radioactive detector). The experimental data reflecting the percentage of unmodified parent compound in plasma vs. time were fitted to the Hill-type function (Equation (1)).

(1) $$fp=d-\frac{\left(d-a\right)t^{b}}{c+t^{b}}$$

where *d* represents the initial level of parent fraction, *a* the plateau, and *b* and *c* the shape modulation factors. Finally, the arterial plasma TACs were corrected by metabolism to represent the plasma concentration of radioactivity corresponding to the parent compound. The corresponding metabolite-corrected arterial plasma TACs were further used for the determination of pharmacokinetic parameters.

#### 2.5.4. Pharmacokinetic Analysis

The pharmacokinetic parameters after intravenous and oral administration at two different doses were calculated by using the add-in program PKSolver. Noncompartmental analysis was used for computing pharmacokinetic parameters of the parent compounds. The terminal slope was automatically estimated using the regression with the largest adjusted *R*^2^. The area under the curve (AUC) was calculated using the log-linear trapezoidal method; other parameters including terminal half-life (*t*_1/2_), mean residence time (MRT), clearance (Cl), volume of distribution based on the terminal slope (*V*_z_) and steady-state volume of distribution (*V*_ss_) were also subsequently calculated. The maximum concentration in plasma (C_max_) and the time taken to reach the maximum plasma concentration (T_max_) were obtained from arterial plasma data. An unpaired two-tailed *t*-test was run to compare the data obtained under no carrier (NCA, administered dose 1 μg/Kg) and under carrier added (CA, administered dose 5 mg/Kg) conditions.

#### 2.5.5. PET Biodistribution Studies

Rats were anesthetized by inhalation of 5% isoflurane (IsoFlo^®^, Abbott Laboratories, Abbott Park, IL, USA) in pure O_2_ and maintained by 1.5–2% isoflurane in 100% O_2_. During imaging, rats were kept normothermic using a heating blanket (Homeothermic Blanket Control Unit; Bruker). For intravenous administration, once the animal was under anaesthesia, one of the lateral tail veins was catheterized with a 24-gauge catheter (Introcan Certo^®^, Bbraun) and [^11^C]AHK2.1 or [^11^C]AHK2.2 was injected using saline solution as the vehicle (*n* = 3 for each radiolabelled compound, 26 ± 5 MBq; dose adjusted to 1 µg/Kg) concomitantly with the start of a PET dynamic acquisition. Whole body dynamic images were acquired in 4 bed positions (21 frames: 5 × 5 s per bed, 5 × 30 s per bed, 5 × 60 s per bed, 6 × 120 s per bed; total acquisition time = 79.6 min) in the 400–700 keV energetic window using an eXploreVista-CT small animal PET-CT system (GE Healthcare, Waukesha, WI, USA). After each PET scan, CT acquisitions were also performed (140 µA intensity, 40 kV voltage) to provide anatomical information of each animal as well as the attenuation map for the later reconstruction of the PET images. PET images were reconstructed using 2D OSEM reconstruction algorithm and applying random, scatter and attenuation corrections. After reconstruction, PET-CT images of the same animal were co-registered and analysed using PMOD image processing tool (PMOD Technologies Ltd., Zürich, Switzerland). Volumes of interest (VOIs) were manually drawn on different organs (brain, lungs, heart, liver, stomach, kidneys, and bladder). Time–activity curves (decay corrected) were obtained as cps/cm^3^ in each organ. Curves were transformed into real activity (Bq/cm^3^) curves by using a calibration factor, obtained from previous scans performed on a phantom (micro-deluxe, Data spectrum Corp.) under the same experimental conditions (isotope, reconstruction algorithm and energetic window) and finally into %ID/cm^3^.

For oral administration, animals were kept in a fasting condition for 5 h before the imaging session. Deep sedation was induced to the animals by inhalation of 5% isoflurane in pure O_2_. As soon as anaesthesia was accomplished, the animals were placed on an angled board at 90° (vertical head up) by hanging the upper incisor teeth on the incisor loop of the board. The radioactive dose (74 ± 10 MBq in 300 μL of saline solution; mass dose adjusted to 1 µg/Kg) was administered using a flexible a 20-gauge disposable flexible PTFE feeding needles (Fine Science Tools, F.S.T.) and a small animal Laryngoscope (Penn-Century, Model LS-2) for the correct visualization of the oesophagus. Administration was followed by whole body static images of 10 min in four bed positions at different time points (0, 30, 60, 90, 120, 160 min) and high-resolution CT acquisitions as above. The time gap between the administration of the labelled compound and the start of the first PET scan was ca. 30 s. After image reconstruction, PET-CT images were analysed using the PMOD image processing tool as above.

## 3. Results

### 3.1. AHK2 Mechanisms of Action

**Figure 2:**
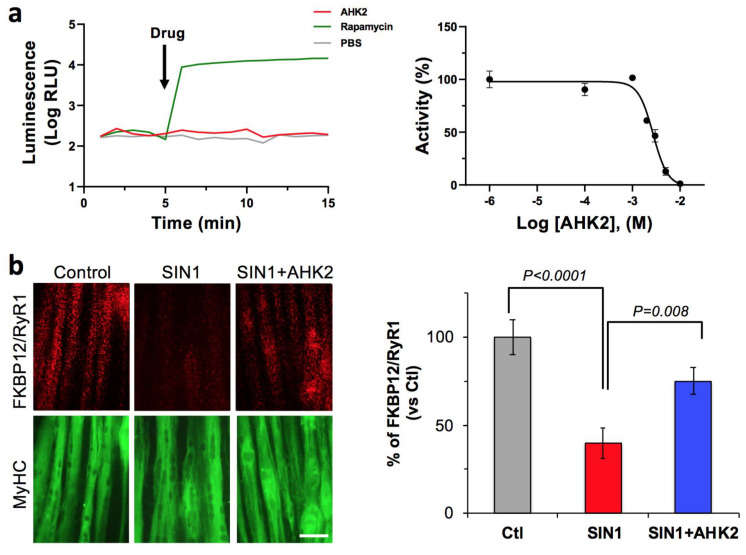
AHK2 compound binds FKBP12 and functions as a RyR/FKBP12 stabilizer. (**a**) Target engagement of AHK2 was evaluated in HEK293 cells using a luminescence protein complementation assay. FKBP12/FRB dimerization was induced with rapamycin, while AHK2 did not induce FKBP12/FRB dimerization (left panel). AHK2 inhibits formation of FKBP12-Rapamycin-FRB interaction with an IC50 value of 2.98 mM (right panel). Graph shows data from a representative experiment repeated three times. Data are expressed as mean + SEM of *n* = 3 replicates. (**b**) AHK2 rescues FKBP12/RyR interaction in human myotubes under nitro-oxidative stress. In situ proximity ligation assay (red) shows that reduced FKBP12/RyR interaction in myotubes challenged with peroxynitrite donor SIN1 (3-morpholino-sydoniminium chloride) is partially rescued by AHK2 with an increase of 89%. Myotubes are counterstained with myosin heavy chain (MyHC, green). Statistical significance was determined using one-way ANOVA followed by Dunnett’s test. *n* = 11–15 images from 3–4 independent experiments. Scale bar: 50 μm.

First, we aimed to verify the target engagement our drug candidate (AHK2) to FKBP12. To do this, we used a well-characterized protein fragment complementation assay where the rapamycin-induced FKBP/FRB interaction generates quantitative luminescence, due to the association of luciferase fragments fused to FKBP12 and the FRB mTOR domain. Indeed, we previously characterized and used this assay to evaluate FKBP12 target engagement of other triazole compounds [19]. In this system, FKBP12 binds rapamycin and, subsequently, the FKBP-rapamycin complex binds FRB to form a ternary complex [20], which results in a drastic increase of luminescence of approximately two orders of magnitude. As expected, AHK2 was not able to induce FKBP12/FRB dimerization, even at high concentrations (10 mM), since it does not present the rapamycin site that binds to FRB (Figure 2a). In contrast, AHK2 competes with rapamycin for the FKBP12 binding pocket, as shown by AHK2 displacement of rapamycin from FKBP12 with a potency (IC50) of 2.98 mM. Next, we wanted to evaluate the activity of AHK2 in modulating the FKBP12/RyR interaction. For this purpose, we used human myotubes subjected to nitro-oxidative stress since FKBP12 depletion of RyR complexes has been previously demonstrated in this system [19]. The FKBP12/RyR interaction was quantified with an in situ proximity ligation assay (in situ PLA) using antibodies specific for RyR and FKBP12 (Figure 2b). Our results indicate that AHK2 shows efficacy in stabilizing the FKBP12/RyR interaction in myotubes under nitro-oxidative stress, with an increase of 89%.

### 3.2. Radiochemistry—^11^C Labelling

Radiolabelling of AHK2 with ^11^C was attempted in two different positions. For the preparation of [^11^C]AHK2.1, in which the [^11^C]CH_3_- moiety is attached to the secondary amine group, the addition of a base was not needed. Our previous experience with ^11^C-radiolabelling of amines [21,22,23] suggested fast reaction rates using the captive solvent method; thus, we initially performed the *N*-methylation at room temperature. As expected, analysis of the reaction crude by radio-HPLC (see ESI; Figure S1b) confirmed the presence of the desired labelled compound [^11^C]AHK2.1 together with a minor peak corresponding to unreacted [^11^C]CH_3_I. Although longer reaction times could lead to higher radiochemical conversion values, the increase in reaction time would also lead to higher radioactivity loss due to decay. Hence, we decided to use these experimental conditions for the fully automated synthesis of [^11^C]AHK2.1. After purification by HPLC (Figure S1c) and reformulation with ethanol and physiologic saline solution (1/9, *v*/*v*) the radiotracer was obtained ready for injection. Quality control by radio-HPLC confirmed a radiochemical purity > 95% and co-elution with the reference standard confirmed the identity of the compound (Figure S2d). Radiochemical yield (non-decay corrected) was 14 ± 2% (*n* = 3) in an overall production time of ca. 30 min from EOB. Molar activities were in the range of 60–110 GBq/µmol.

The synthesis of [^11^C]AHK2.2 required the attachment of the [^11^C]CH_3_- moiety to the phenol group, and hence the use of a strong base to generate the nucleophile in situ was required. For initial runs, NaOH was considered as the base as this is routinely used in our laboratory for the preparation of [^11^C]Raclopride [18], a radiotracer in which the [^11^C]CH_3_- moiety is also attached to the phenol group. The addition of 3 µL of aqueous 1M NaOH solution as the base led to the formation of the desired compound in a 5-minute reaction time at room temperature, with radiochemical conversion values close to 40% (See ESI; Figure S2b). The presence of significant amounts of [^11^C]CH_3_OH as side-product together with unreacted [^11^C]CH_3_I suggested that other bases may lead to better results. Hence, we decided to investigate sodium hydride (NaH; dispersion in mineral oil) using DMF as the reaction solvent. Analysis of the crude reaction product (Figure S2b) showed almost quantitative chromatographic yield under these experimental conditions. No unreacted [^11^C]CH_3_I was detected, and only the presence of two very minor unidentified peaks could be observed in the chromatogram. After purification by HPLC (Figure S2c) and reformulation with ethanol and physiologic saline solution (1/9, *v*/*v*), the radiotracer was obtained ready for injection. Quality control by radio-HPLC confirmed a radiochemical purity > 95% and co-elution with the reference standard AHK2 confirmed the identity of the compound. Radiochemical yield (non-decay corrected) was 15 ± 2% (*n* = 3) in overall production time of ca. 30 min from EOB. Molar activities were in the range of 68–122 GBq/µmol.

### 3.3. Radiochemistry—^3^H Labelling

Different tritiation methods have been described in the literature [24]. From among them, we selected the catalytic halogen-tritium replacement method. Upon reaction completion and purification, the radioactive chromatogram showed the presence of a single peak (see ESI; Figure S3c) that co-eluted with the reference standard (Figure S3b). The isotopic incorporation was determined by mass spectrometry to be 54.1% unlabelled and 45.9% mono-labelled (Figure S3d) to give a molar activity of 487 KBq/nmol.

### 3.4. In Vitro Metabolism

**Figure 3:**
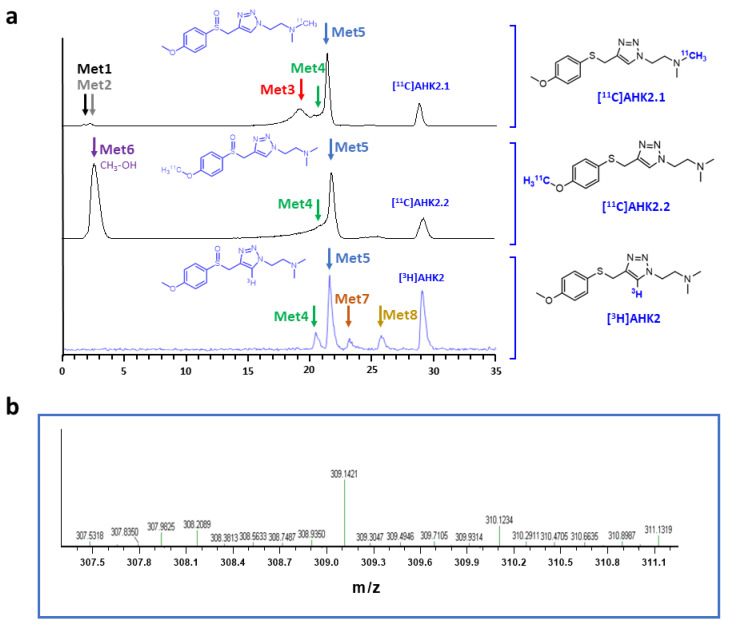
(**a**) Representative chromatograms corresponding to the in vitro metabolism (radioactivity detector) of [^11^C]AHK2.1 (top), [^11^C]AHK2.2 (middle) and [^3^H]AHK2 (bottom). The position of radioactive metabolites with the same retention times are indicated with colour arrows; (**b**) mass spectrum corresponding to peak indicated with a blue arrow in 2a, conforming the formation of the sulfoxide derivative.

The formation of metabolites was first investigated in vitro to have an estimation of metabolic rates and to potentially identify metabolic routes. Analysis via HPLC of the incubation media containing [^11^C]AHK2.1 (Figure 3a) showed the presence of the unmodified parent compound (retention time = 29.2 min) and the formation of two very minor polar metabolites with retention times of ca. 1.8 and 2.4 min (Met1 and Met2; Figure 3a, displayed in black and grey, respectively). Additionally, three non-polar, poorly resolved radioactive metabolites were found with retention times = 19.5, 20.3 and 21.2 min (Met3, Met4 and Met5; Figure 3a, displayed in red, green, and blue, respectively). Moving to the compound [^11^C]AHK2.2, the radio-HPLC analysis showed the presence of two major peaks, one of them with strong polarity and short retention time (Met6; retention time = 2.5 min) and the other corresponding to Met5 (retention time = 21.2 min). The presence of a left-shoulder in the latter peak suggests the presence of Met4 (Figure 3a). Finally, analysis of the chromatogram obtained for [^3^H]AHK2 (Figure 3a) showed the formation of four non-polar radioactive metabolites, with retention times 20.3, 21.2, 22.5 and 25.5 min (Met4, Met5, Met7 and Met8; displayed in green, blue, orange and yellow, respectively).

**Figure 4:**
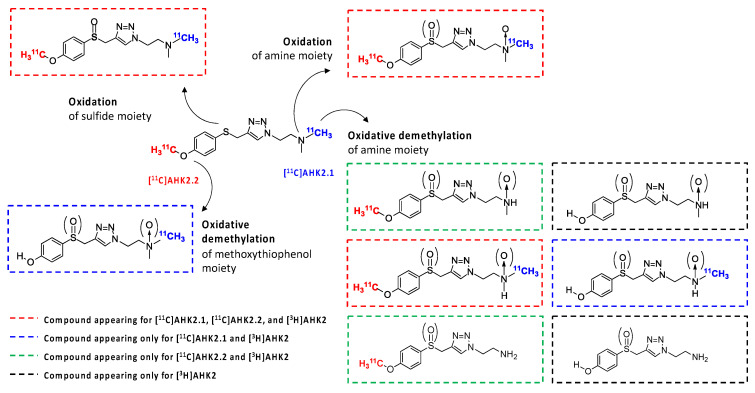
Hypothesized metabolic route of AHK2 based on chromatograms shown in 3a and HR-MS analysis.

Besides the parent compound, the only major peak appearing in all three chromatograms was that with retention time = 21.2 min (Met 5). To confirm the identity of this metabolite, we incubated the reference compound AHK2 with rat liver microsomes and analysed the resulting mixture by LC-MS. Mass spectrometry analysis suggested the presence of the sulfoxide derivative with *m*/*z* = 309.14 (M-H^+^; Figure 3b) together with minor amounts of the isobaric N-oxide (see Figure S12), which was further confirmed by co-elution with reference sulfoxide derivative (results not shown). The presence of this major metabolite confirms that the first metabolic step is probably the oxidation of the sulfide moiety. Interestingly, the presence of a peak corresponding to a very polar labelled compound for [^11^C]AHK2.2 suggests the formation of [^11^C]CH_3_OH, resulting from oxidative demethylation (Figure 4). The presence of two peaks with short retention times in the case of [^11^C]AHK2.1 suggests that the metabolic pathway also goes via oxidative demethylation of the amine group (resulting in the formation of [^11^C]CH_3_OH, which would correspond to Met2 in Figure 3a). The loss of [^11^C]CH_3_OH and cleavage of the alkyl chain of the triazole ring would explain the presence of non-polar radioactive metabolites that are visible only for [^3^H]AHK2.

### 3.5. In Vivo Metabolism

In vivo metabolism was subsequently investigated in rats. In this case, the presence of metabolites was investigated at different time points after administration of both [^11^C]AHK2.1 and [^11^C]AHK2.2. The analysis of the plasmatic fraction after the intravenous administration of [^11^C]AHK2.1, showed the presence of two metabolites with short retention times, already visible at t = 2 min post administration, that progressively increase over time to reach values of ca. 70% of the total radioactivity (both together) at 15 min after administration (see ESI, Figure S4a). The short retention time confirms the high polarity of the metabolites and suggests that, as observed in vitro, the metabolic pathways go via oxidative demethylation of the amine group, resulting in the formation of [^11^C]CH_3_OH) and/or cleavage of the alkyl chain of the triazole ring. Two further metabolites, with retention times pf ca. 21 and 22 min, respectively, were also observed, particularly at t = 5 min after administration of the compound. The peak at t = 21 min may correspond to two different metabolites (Met3 and Met4 detected also in vitro), while the peak at t = 22 min probably corresponds to Met5, resulting from oxidation of the sulfide moiety. Interestingly, the relative concentration of these metabolites (Met3–5) progressively decreases after peaking at t = 5 min post injection, suggesting that these species are further metabolised or eliminated. At t = 15 min post administration, the presence of the parent compound is testimonial.

Analysis of the plasmatic fraction after the intravenous administration of the labelled compound [^11^C]AHK2.2 showed the presence of one peak with strong polarity and short retention time (t = ca. 2 min; see Figure S4b), suggesting the formation of [^11^C]CH_3_OH as the major metabolite. Two further metabolites with retention times of ca. 21 and 22 min, respectively, were also observed, probably corresponding to metabolites 4 and 5, respectively. The presence of the major metabolite with the retention time = ca. 22 min, which progressively increases in intensity to later on decrease for both [^11^C]AHK2.1 and [^11^C]AHK2.2, strengthens the hypothesis of the formation of the sulfoxide derivative, which is followed by oxidative demethylation and demethylation of the amine group and/or cleavage of the alkyl chain of the triazole ring.

After oral administration, radio-HPLC analysis of arterial blood samples showed the same metabolic pathway already observed after the intravenous administration, although signal to noise ratio was compromised due to the lower concentration of radioactivity in blood. When [^11^C]AHK2.1 was administered, the analysis of the plasmatic fraction showed the presence of four radioactive metabolites with retention times = 2, 3, 21 and 22 min, respectively (Figure S5a). When compound [^11^C]AHK2.2 was administered, the formation of three radioactive metabolites with retention times = 2, 21 and 22 min, respectively, could be detected (Figure S5b).

### 3.6. Time-Activity Curves and Pharmacokinetic Analysis

The extracorporeal circulation of arterial blood enabled the determination of real-time TACs showing the concentration of radioactivity in arterial blood after intravenous and oral administration of the compound at two different concentrations: 1 µg/Kg and 5 mg/Kg. As the concentration of parent compound in arterial plasma is independent of the labelling position, we decided to perform the experiments using only [^11^C]AHK2.1.

**Figure 5:**
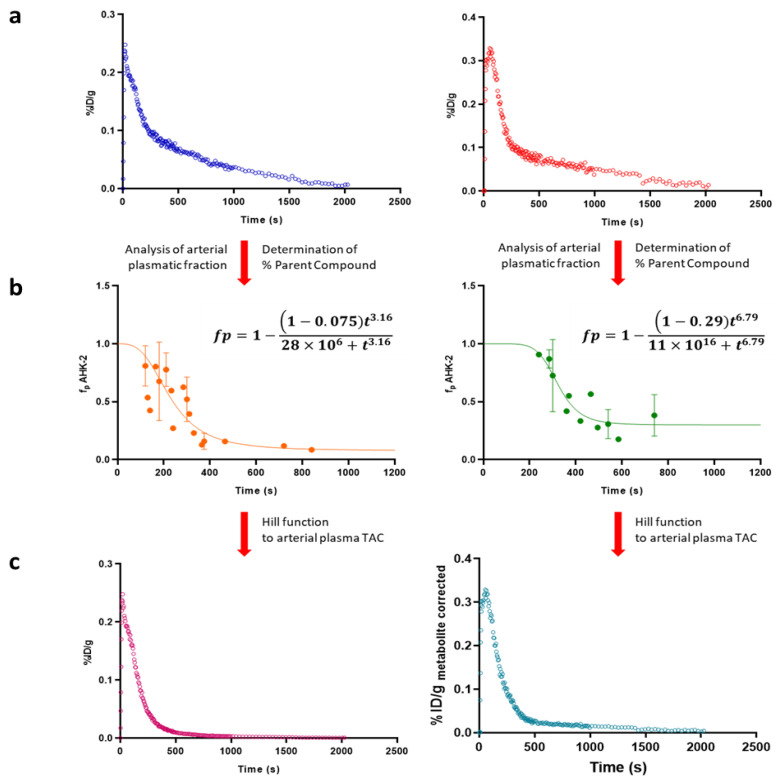
(**a**) Representative arterial plasma Time Activity Curves (TACs) after intravenous administration of [^11^C]AHK2.1 at [1 μg/Kg] (left) and [5 mg/Kg] (right); (**b**) graphic representation of plasma concentration over time of AHK2 after intravenous administration of ^11^C-labelled AHK2 at [1 μg/Kg] (left) and [5 mg/Kg] (right). The experimental values of unmetabolized parent compound (dots in the figure) were fitted using Hill type function (line) to obtain a continuous time course of plasma concentration of parent compound; (**c**) metabolite-corrected arterial plasma TACs after intravenous administration of ^11^C-labelled AHK2 at [1 μg/Kg] (left) and [5 mg/Kg] (right).

In order to obtain arterial plasmatic TACs (see Figure 5a for intravenous administration; Figure S6a for oral administration), arterial blood TACs were corrected by *C_p_*/*C_b_* ratio, which was determined as 1.44 ± 0.2, resulting in a percentage of radioactivity in plasma of ca. 81% (haematocrit values = 0.44 ± 0.04). Experimental values corresponding to unmetabolized parent compound obtained from metabolism studies were fitted using a Hill type function to obtain a continuous time course of plasma concentration of parent compound.

After intravenous administration, optimal fit of the Hill function was obtained when *d* = 1, which means that the compound is not metabolized before the blood reaches the collection site. Values for *a* were 0.075 and 0.29 (dose = 1 μg/Kg and 5 mg/Kg, respectively) (Figure 5b). After oral administration, a lower initial plasma concentration of radioactivity was obtained, as expected (Figure S6a). As for intravenous administration, the best-fit value for the Hill-type function for parameter *d* was 1. However, in the case of oral administration no significant differences were found for the parameter *a* when 1 μg/Kg or 5 mg/Kg were administered, with values of 0.10 and 0.13, respectively.

Hill functions were used to correct arterial plasmatic TACs into metabolite-corrected TACs (Figure 5c and Figure S6c for intravenous and oral administration, respectively), finally used to estimate pharmacokinetic parameters, which were computed by noncompartmental analysis using the curve stripping technique by the add-in program PKSolver [25]. An unpaired two-tailed *t*-test was run to compare the non-carrier added (NCA; administered dose = 1 µg/kg) and the carrier added (CA; administered dose = 5 mg/kg) groups (confidence level = 95%).

No significant differences on pharmacokinetics were found when doses of 1 μg/Kg and 5 mg/Kg were used (Table 1), indicating that the pharmacokinetics is linear in this dose range (*p* values > 0.05). Because of that, all data (irrespective of the administered dose) were pooled.

For oral administration, the pharmacokinetic parameters were calculated by non-compartmental analysis of metabolite-corrected arterial plasma TACs with extravascular input. No significant differences on pharmacokinetics were found when doses of 1 μg/Kg and 5 mg/Kg were used, indicating that the pharmacokinetics is linear in this dose range. Interestingly, pharmacokinetic parameters obtained after oral administration are quite similar to those obtained after intravenous administration for both compounds. This is expected considering that once absorbed, the unaltered compound undergoes the same processes as when administered intravenously. The only differences are observed for AUC values. The AUC, which describes the total amount of unaltered drug that reaches the systemic circulation, is the most reliable measure of a drug’s bioavailability. The bioavailability (F) of a drug delivered intravenously is theoretically 100% (F = 1), therefore the bioavailability of a drug administered by oral route can subsequently be determined by comparing the respective AUCs (F = AUC_oral_/AUC_i.v._, if the same dose is received by each route). The bioavailability after oral administration was 0.185 (Table 2).

### 3.7. In Vivo Pet Imaging

**Figure 6:**
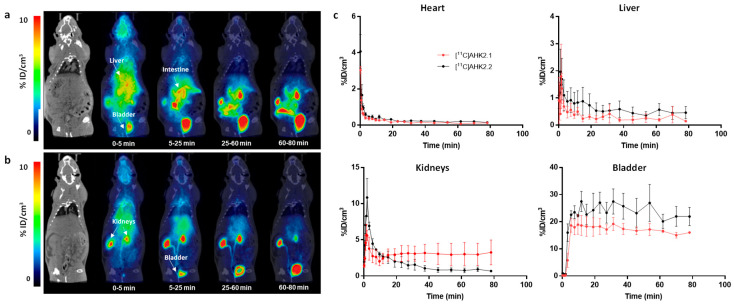
Biodistribution of [^11^C]AHK2.1 (**a**) and [^11^C]AHK2.2 (**b**) after intravenous administration (dose = 1 μg/Kg). Representative whole body coronal PET images (projections) obtained at different time points after administration. PET images have been co-registered with a representative CT slice for the anatomical localization of the radioactive signal. Positions of the major organs are indicated. Scale bar in in Standard Uptake Value (SUV) units; (**c**) concentration of radioactivity in the different organs as determined from PET images after intravenous administration of [^11^C]AHK2.1 and [^11^C]AHK2.2. Values are expressed as %ID/cm^3^. Values correspond to mean ± standard deviation, *n* = 3 per radiolabelled compound.

PET imaging was finally performed to gain knowledge about the biodistribution of both [^11^C]AHK2.1 and [^11^C]AHK2.2 after intravenous and oral administration. Visual inspection of dynamic PET images obtained after intravenous administration of [^11^C]AHK2.1 majorly reflected accumulation in the gastrointestinal tract, the kidneys and the bladder (Figure 6a), while administration of [^11^C]AHK2.2 resulted in rapid accumulation of radioactivity in the kidneys and elimination via urine (Figure 6b). The difference observed among the two labelled compounds is due to the different major metabolites formed after administration. Delineation of volumes of interest (VOIs) in major organs (brain, heart, lungs, liver, kidneys, and bladder) and determination of the uptake as % of injected dose per cm^3^ (%ID/cm^3^, see Figure 6c for selected organs) revealed a higher retention of radioactivity in the kidneys after administration of [^11^C]AHK2.1, while kidney washout of [^11^C]AHK2.2 was faster resulting in a higher elimination via urine. Unfortunately, delineation of VOIs in the small and large intestines was not possible based on anatomical information provided by CT images, and direct quantitative comparison was not possible. The different biodistribution patterns observed when [^11^C]AHK2.1 or [^11^C]AHK2.2 are administered suggest that metabolism studies are paramount to interpret PET images.

For oral administration, visual inspection of PET images (Figure S7) showed no translocation to remote organs for both compounds indicating that the absorption was too low to be measured by PET imaging. This could be a result of the low bioavailability of the compound, together with the short half-life of the radionuclide, which dramatically decreases the sensibility of the technique at long times after administration.

## 4. Discussion

The determination of the pharmacokinetic properties of new chemical entities is paramount before entering clinical phases. Radiolabelling strategies often combined with nuclear imaging are progressively being incorporated into the drug development pipeline, as the detection of radioactive substances can usually be achieved with high sensitivity.

The first key step in the application of radiolabelling/nuclear imaging to the investigation of pharmacokinetic properties of a new chemical entity is the selection of the radionuclide. Ideally, the chemical structure of the target molecule should not be altered, and hence “formal replacement” of a stable atom already present in the molecule under investigation by a radioisotope of the same element needs to be considered. The vast majority of organic molecules contain carbon, hydrogen and nitrogen atoms, and hence labelling strategies using the positron emitters nitrogen-13 [26] or carbon-11 [27,28], or the low energy beta emitters hydrogen-3 or carbon-14 can be applied to obtain radiolabelled molecules with exactly the same chemical structure of the compound under investigation. The main drawback with the positron emitters is the short half-life of both ^11^C and ^13^N (20.4 and 9.97 min, respectively) which may not be sufficient to track the labelled compound (or its metabolites) over the whole residence time in the body. Contrarily, both ^3^H and ^14^C have very long half-lives, although they are not suitable for imaging purposes.

After selection of the radionuclide/s, the second key decision is the labelling position. Analytical tools or imaging devices detect the presence of radioactivity. In other words, if the molecule metabolises, only those metabolites containing the radionuclide and the parent compound will be detected. Consequently, labelling with one radionuclide in one position may offer just a piece of the whole puzzle, while a combination of radionuclides and labelling sites can offer complementary information.

In this work, we decided to combine two radionuclides and three labelling positions to gain a wide perspective on the pharmacokinetic behaviour of the chemical species under investigation. Importantly, we used an extracorporeal circulation system to have a fully resolved time-activity curve. The determination of the concentration of parent compound in plasma is usually carried out in discrete blood samples obtained at pre-defined time-points. In our case, continuous measurement of the concentration of radioactivity in blood, combined with metabolite analysis in discrete samples and determination of C_P_/C_b_ ratio allowed for the generation of arterial plasma TACs, which is very convenient for model fitting and extraction of pharmacokinetic parameters.

Our study has certain limitations. First, it is worth mentioning that the analysis of metabolites just detects radiolabelled metabolites present in the plasma and the percentage of radioactivity that remains as unmodified parent compound. However, identification of the chemical structure of the metabolites requires structural analytical techniques, such as LC-MS. In our study, only one of the metabolites could be unambiguously identified, although metabolic pathways could be hypothesized due to the complementary information gathered from the different experiments.

The second limitation of the study is related to the determination of the arterial TAC. The most intuitive approach to determine the arterial TAC consists of withdrawing discrete arterial blood samples from the investigated subject, which are then analysed to determine the concentration of the unmodified parent compound. This process has major drawbacks: (i) it offers discrete information for pre-selected time points and (ii) it is relatively invasive, as it requires arterial blood samples; additionally, and particularly in the context of small rodents, such as mice and to a lesser extent rats, it has the inconvenience of withdrawing large blood volumes, which may induce physiological alterations and compromise the health status of the animal. Venous blood-based input functions offer a reasonable (and less invasive) alternative. However, this alternative also has limitations: first, the input function corresponds to venous blood, which is less appropriate than arterial blood to extract pharmacokinetic parameters. Additionally, the relation between arterial and venous input functions changes with time and may show inter-subject variability [29]. To overcome these limitations, and after radiolabelling with a positron emitter, the TAC can be derived from acquired images [30]. This approach relies on the quantification of the concentration of radioactivity in blood by drawing, directly on the images and a VOI in the blood pool, i.e., an artery, a vein of the left ventricle. This approach is not invasive and does not alter the physiology of the animal, although it requires a correct visualization of the vascular space (usually challenging in the preclinical setting) and the values obtained can be affected by spill-over and partial volume effect [31,32]. The last alternative to obtain arterial TAC in the preclinical setting, which is probably the most laborious but also accurate method and is the one used here, consists of generating an extracorporeal circulation by-pass from one of the arteries to one of the veins of the animal [33]. This can be combined with discrete blood sampling and subsequent analysis. This approach provides continuous arterial Ifs which can be corrected for metabolism. Still, major disadvantages include the time-consuming animal preparation process and its inherent invasiveness, which hamper the reuse of the animals in longitudinal studies. Additionally, the systematic time difference between the arrival times of the radiolabelled compound in the tissue relative to the peripheral sampling site (outside of the animal) and the smearing of the shape of the TAC, as the tracer transport within the tube system is influenced by its first order lag kinetics, lead to dispersion of the TAC. Some methods to correct for dispersion have been reported [34], although they are specific for each experimental configuration and have not been applied in our study. Hence, our extracorporeal determined arterial TACs probably show a deviation with respect to real arterial TACs, which may induce a bias in the determination of pharmacokinetic parameters.

## 5. Summary and Conclusions

In this work, we have developed efficient radiolabelling strategies for the incorporation of either positron (^11^C) or beta (^3^H) emitters into the triazole-based FKBP12-RyR stabilizer AHK-2. ^11^C-labelling was achieved by ^11^C-methylation using [^11^C]CH_3_I as labelling agent. The labelling with beta emitters was achieved by reduction of the correspondent 5-iodotriazole derivative using tritium gas. The multi-radionuclide/multi-position labelling approach resulted in being useful to obtain a good overview of metabolism and the identification of some of the metabolites. Preliminary in vitro metabolism studies, by using rat liver microsomes, suggested that the main metabolic pathway was oxidation of the sulfide group into sulfoxide and tertiary amine into N-oxide, together with the demethylation of the CH_3_O-Ar residue and the N(CH_3_)_2_ group.

The online measurement of the arterial TAC by creating an extracorporeal circulation of the arterial blood allowed for the determination of a real arterial input function and enabled arterial blood sample collection and processing (eventually enabling metabolite analysis) and subsequent correction. For both administration routes (intravenous and oral), the pharmacokinetics were best described by a non-compartmental model. The results indicate high plasma clearance, linear pharmacokinetics in the dose-range investigated (1 µg/Kg and 5 mg/Kg) and low bioavailability when the drug was administrated orally. Investigation of the biodistribution patterns after intravenous administration of the radiolabelled compounds show different pattern depending on the labelling position, suggesting that knowledge about metabolic pathways is paramount to interpret images.

## Figures and Tables

**Table 1:** Main pharmacokinetic parameters of AHK2 obtained from metabolite-corrected arterial plasma time activity curves after intravenous administration to Sprague Dawley Rats.

Entry	Parameter	Value (1 µg/Kg)	Value (5 mg/Kg)	*p* Value	Value *
1	t_1/2_	11.67 ± 3.22	13.60 ± 3.42	0.38	12.63 ± 3.29
2	Cl	1.91 ± 0.41	1.87 ± 0.73	0.71	1.89 ± 0.55
3	V_z_	1996.50 ± 429.72	2335.03 ± 608.77	0.66	2165.76 ± 1125.06
4	V_ss_	1026.88 ± 695.33	1126.13 ± 443.15	0.75	1076.50 ± 808.72
5	AUC	53.99 ± 11.04	59.39 ± 19.76	0.6	56.69 ± 15.36
6	MRT	8.48 ± 3.76	8.59 ± 5.23	0.67	8.53 ± 4.30

t_1/2_: terminal half-life, expressed in minutes; Cl: plasma clearance, expressed in mL/s; V_z_: volume of distribution based on the terminal slope, expressed in mL; Vss: volume of distribution at the steady state, expressed in mL; AUC: area under the curve, expressed as %IDxs/mL; MRT: mean residence time, expressed in minutes. Values are expressed as mean ± standard deviation, *n* = 5 per group. * Considering both groups (1 µg/Kg and 5 mg/Kg) pooled together.

**Table 2:** Main pharmacokinetic parameters of AHK2 obtained from metabolite-corrected arterial plasma time activity curves after oral administration to Sprague Dawley Rats.

Entry	Parameter	Value (1 µg/Kg)	Value (5 mg/Kg)	*p* Value	Value *
1	t_1/2_	9.16 ± 3.78	12.97 ± 11.06	0.6	11.34 ± 8.44
2	Cl	4.23 ± 3.58	1.57 ± 0.69	0.29	2.71 ± 2.55
3	V_z_	2728.14 ± 1252.35	1624.28 ± 1299.35	0.31	2097.36 ± 1309.61
4	AUC	6.68 ± 4.35	13.35 ± 5.08	0.32	10.49 ± 5.65
5	MRT	8.51 ± 2.69	9.36 ± 8.51	0.88	9.00 ± 6.93
6	F				0.185

t_1/2_: terminal half-life, expressed in minutes; Cl: plasma clearance, expressed in mL/s; V_z_: volume of distribution based on the terminal slope, expressed in mL; Vss: volume of distribution at the steady state, expressed in mL; AUC: area under the curve, expressed as %IDxs/mL; MRT: mean residence time, expressed in minutes. Values are expressed as mean ± standard deviation, *n* = 3 per group. * Considering both groups (1 µg/Kg and 5 mg/Kg) pooled together.

## Data Availability

The datasets used and/or analysed during the current study are available from the corresponding author on reasonable request.

## Supplementary Materials

The following supporting information can be downloaded at: https://www.mdpi.com/article/10.3390/biomedicines11020253/s1, Figure S1: Reaction scheme for the production of [^11^C]AHK2.1 and chromatographs obtained for the reaction crude and during purification; Figure S2: Reaction scheme for the production of [^11^C]AHK2.2 and chromatographs obtained for the reaction crude and during purification; Figure S3: Reaction scheme for the production of [^3^H]AHK2, chromatographs obtained during purification and mass spectrum of [^3^H]AHK2; Figure S4: chromatograms corresponding to the evaluation of the presence of metabolites in plasma after intravenous administration of [^11^C]AHK2.1 and [^11^C]AHK2.2 at 1 μg/Kg; Figure S5: chromatograms corresponding to the evaluation of the presence of metabolites in plasma after oral administration of [^11^C]AHK2.1 and [^11^C]AHK2.2 at 1 μg/Kg; Figure S6: Representative arterial plasma Time Activity Curves after oral administration of [^11^C]AHK2.1 at different doses, plasma concentration of AHK2 after intravenous administration, and metabolite-corrected arterial plasma time-activity curves after intravenous administration of ^11^C-labelled AHK2; Figure S7: Representative PET images obtained after oral administration of [^11^C]AHK2.1 and [^11^C]AHK2.2; Figures S9–S12: NMR spectra of the different compounds and intermediates.
