# Supplementary material for: Pharmacokinetic Evaluation of New Drugs Using a Multi-Labelling Approach and PET Imaging: Application to a Drug Candidate with Potential Application in Neuromuscular Disorders
Source: Biomedicines. 2023 Jan 18;11(2):253. doi: 10.3390/biomedicines11020253 (PMC9953224; doi:10.3390/biomedicines11020253)

TITLE: Pharmacokinetic evaluation of new drugs using a multi-labelling approach and PET imaging: application to a drug candidate with potential application in neuromuscular disorders.

Authors' full names: Rossana Passannante,[1] Vanessa Gómez-Vallejo,[1] Maialen Sagartzazu-Aizpurua,[2] Laura Vignau,[2] Pablo Marco-Moreno,[3,4] Garazi Aldanondo,[4] Ainara Vallejo-Illarramendi,[3,4] Pablo Aguiar,[5] Unai Cossío,[1] Abraham Martín,[6,7] Jonas Bergare,[8] Lee Kingston,[8] Charles S. Elmore,[8] Miguel Angel Morcillo,[9] Pablo Ferrón,[10] Jesus M. Aizpurua,[2] Jordi Llop,[1,*]

Authors' affiliation(s):

[1] CIC biomaGUNE, Basque Research and Technology Alliance (BRTA), 20014-San Sebastián, Spain

[2] Departamento de Química Orgánica-I, UPV/EHU-University of the Basque Country, 20018-San Sebastián, Spain

[3] Group of Neuroscience, Department of Pediatrics, Hospital Donostia, UPV/EHU, 20014-San Sebastián, Spain

[4] Group of Neuromuscular Diseases. Biodonostia Health Research Institute, 20014-San Sebastián, Spain

[5] Molecular Imaging Group, IDIS, CIMUS, Universidad de Santiago de Compostela, Spain

[6] Ikerbasque, Basque Foundation for Science, Maria Diaz de Haro 3, 48013-Bilbao, Spain.

[7] Laboratory of Neuroimaging and biomarkers of inflammation, Achucarro Basque Center for Neuroscience, Science Park UPV/EHU, Sede building B, Sarriena, 48940-Leioa, Spain

[8] Early Chemical Development, Pharmaceutical Sciences R&D, AstraZeneca, 431 83-Gothenburg, Sweden

[9] CIEMAT, Medical Applications of Ionizing Radiations Unit, Madrid, Spain

[10] Miramoon Pharma S.L., Avda Tolosa-72, 20018-San Sebastián, Spain

Corresponding author details:

Name: Jordi Llop

Email: jllop@cicbiomagune.es

Figure S1. a) Reaction scheme for the production of [^{11}C]AHK2.1; b) Chromatograph (radioactivity detector) obtained after analysis of the reaction crude in the production of [^{11}C]AHK2.1; c) Chromatograph (radioactivity detector) obtained during purification of the reaction crude in the production of [^{11}C]AHK2.1. The peak corresponding to [^{11}C]AHK2.1 (green area) was collected and reformulated.

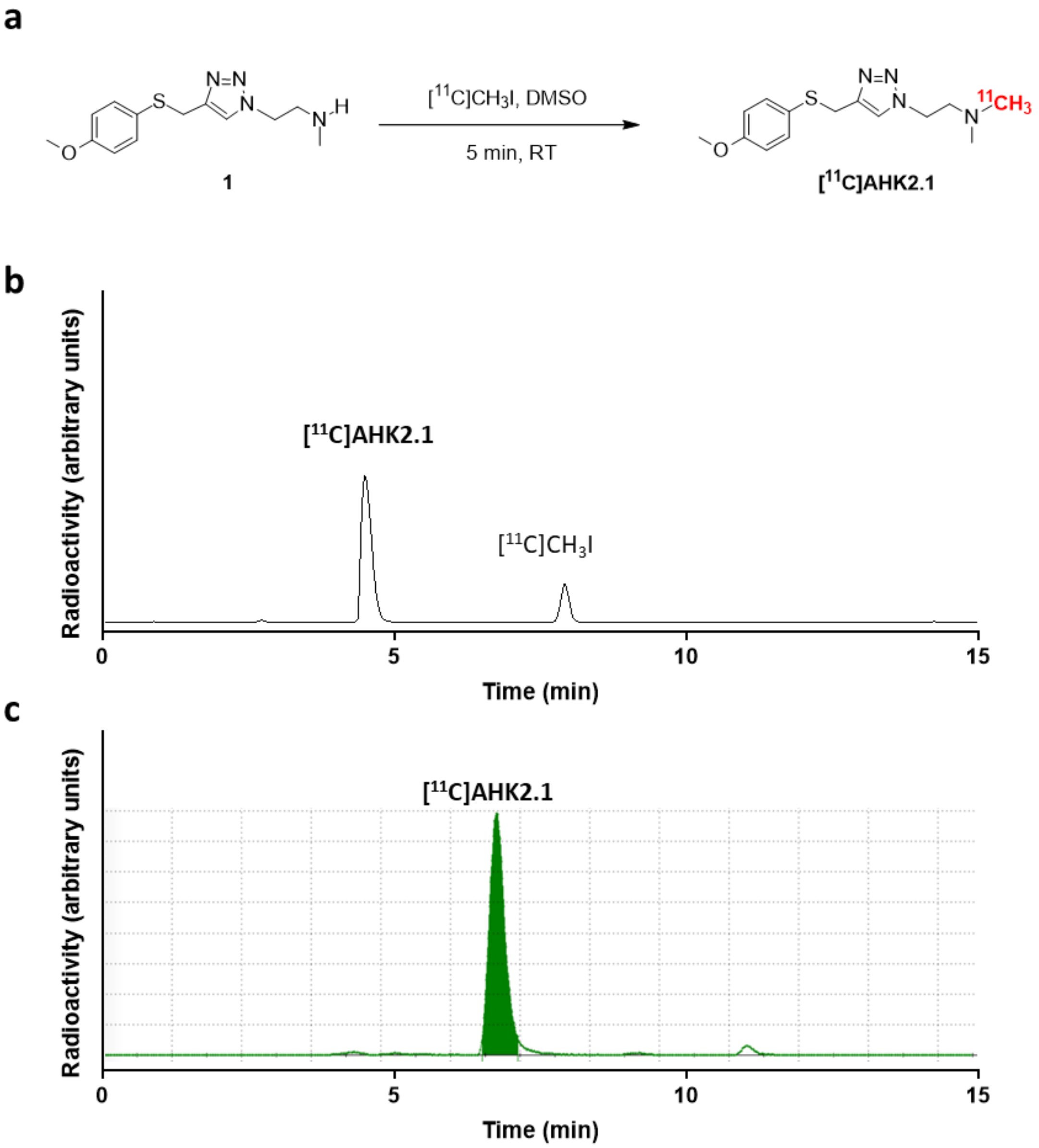

Figure S2. a) Reaction scheme for the production of [^{11}C]AHK2.2; b) Chromatographs (radioactivity detector) obtained after analysis of the reaction crude in the production of [^{11}C]AHK2.2 using NaOH (top) and NaH (bottom) as the base; c) Chromatograph (radioactivity detector) obtained during purification of the reaction crude in the production of [^{11}C]AHK2.2. The peak corresponding to [^{11}C]AHK2.2 (green area) was collected and reformulated.

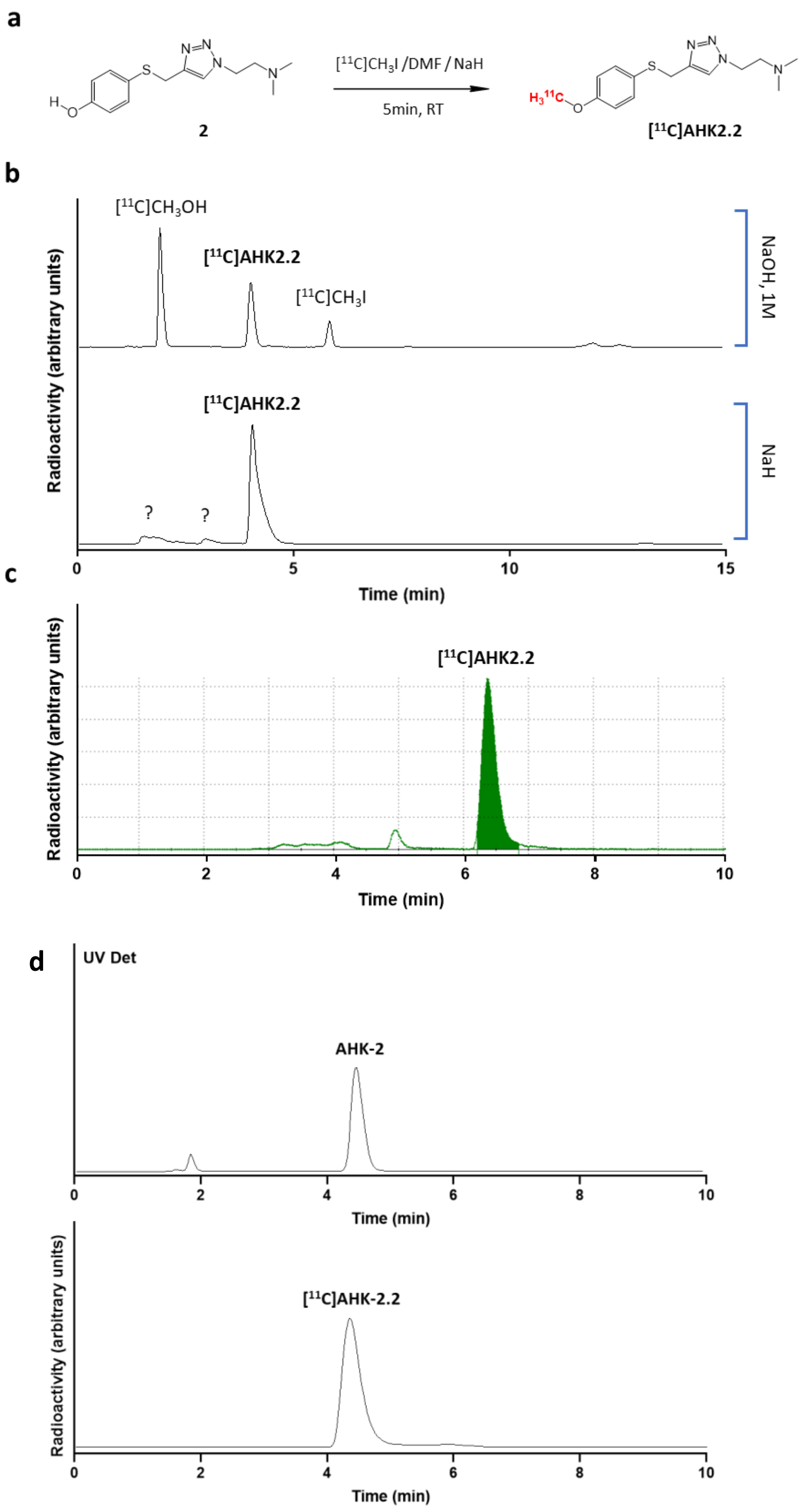

Figure S3. a) Reaction scheme for the production of [^{3}H]AHK2; b,c) Chromatographs (UV and radioactivity detectors, respectively) obtained after purification of [^{3}H]AHK2; d) mass spectrum of the indicated chromatographic peak, confirming the formation of [^{3}H]AHK2.

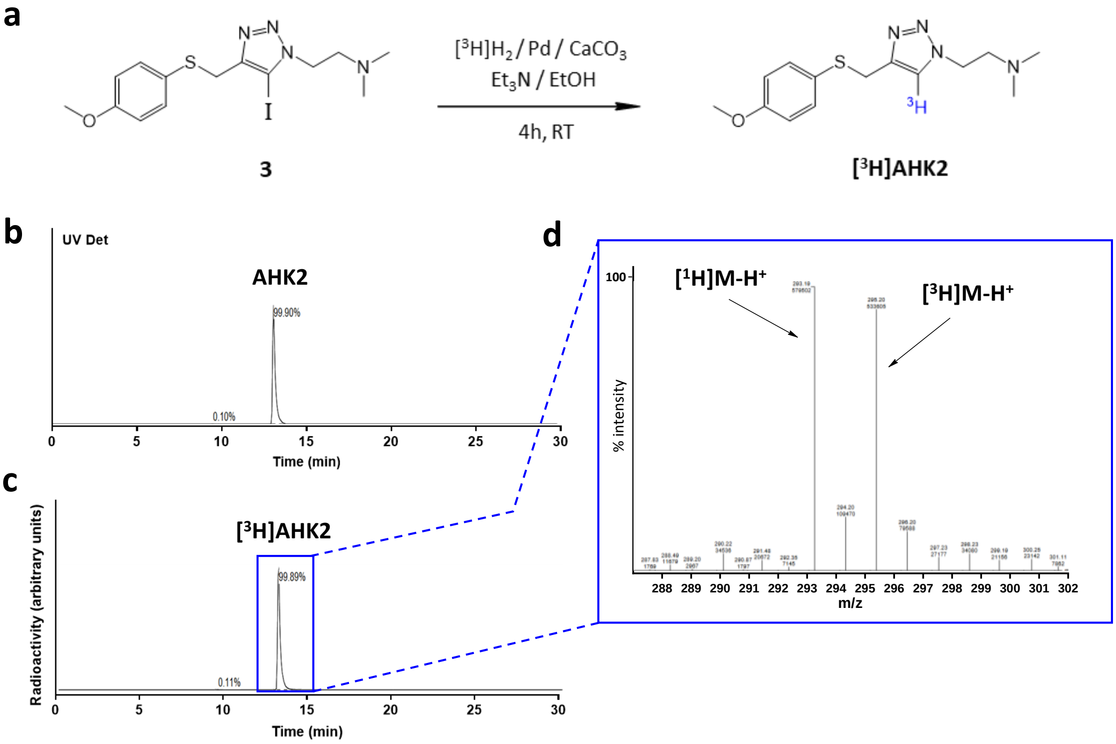

Figure S4. Representative chromatograms (radioactivity detector) corresponding to the evaluation of the presence of metabolites in plasma after intravenous administration of [^{11}C]AHK2.1 (a) and [^{11}C]AHK2.2 (b) at injected dose = 1 μg/Kg. The position of the peaks corresponding to major metabolites are shown.

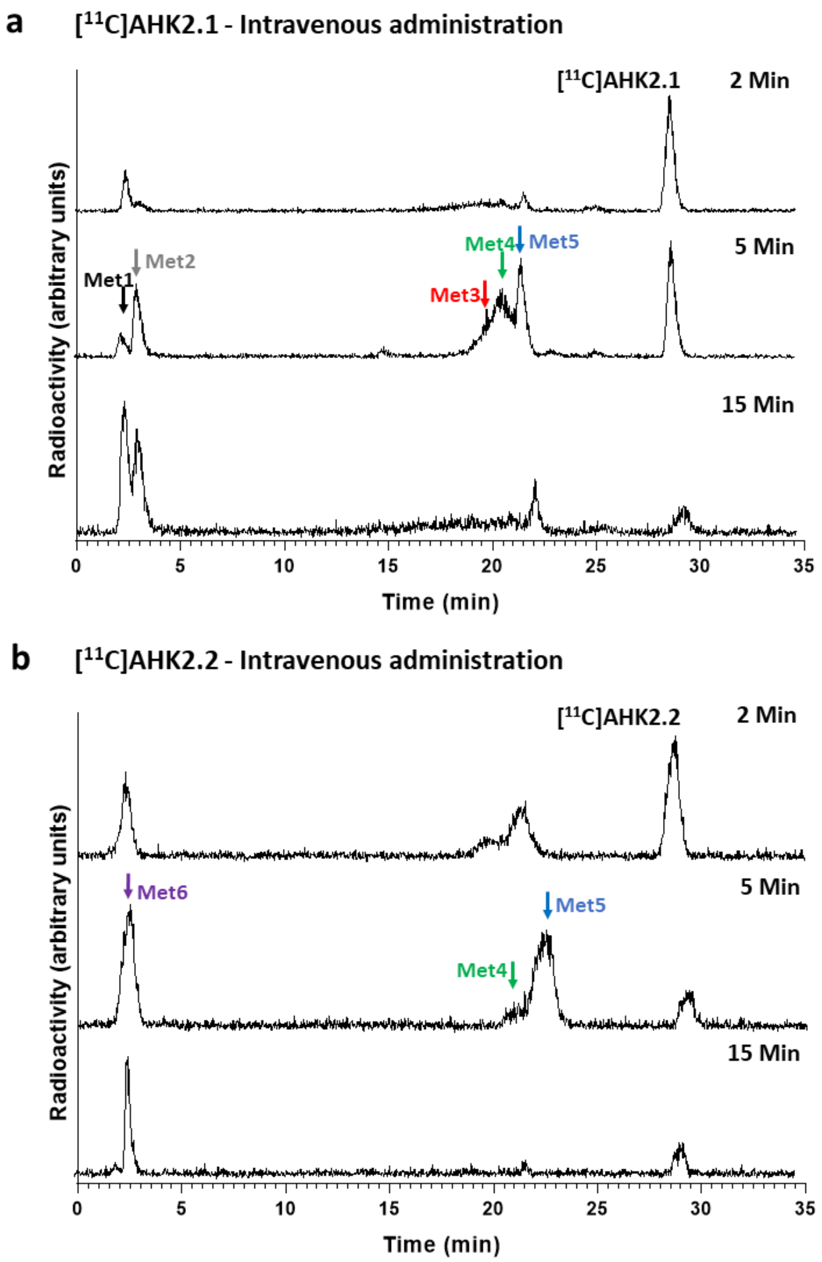

Figure S5. Representative chromatograms (radioactivity detector) corresponding to the evaluation of the presence of metabolites in plasma after oral administration of [^{11}C]AHK2.1 (a) and [^{11}C]AHK2.2 (b) at injected dose = 1 µg/Kg. The position of the peaks corresponding to major metabolites are shown.

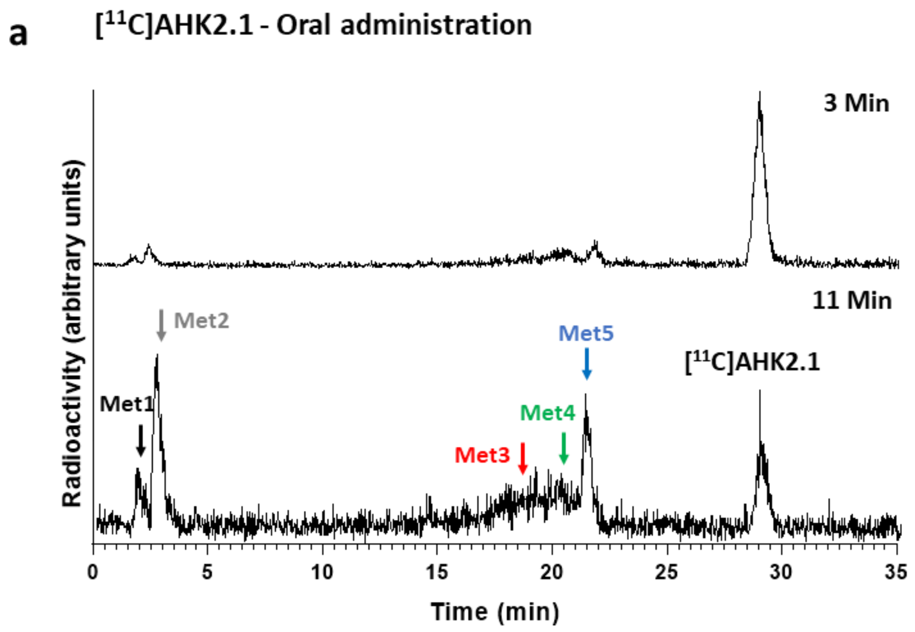

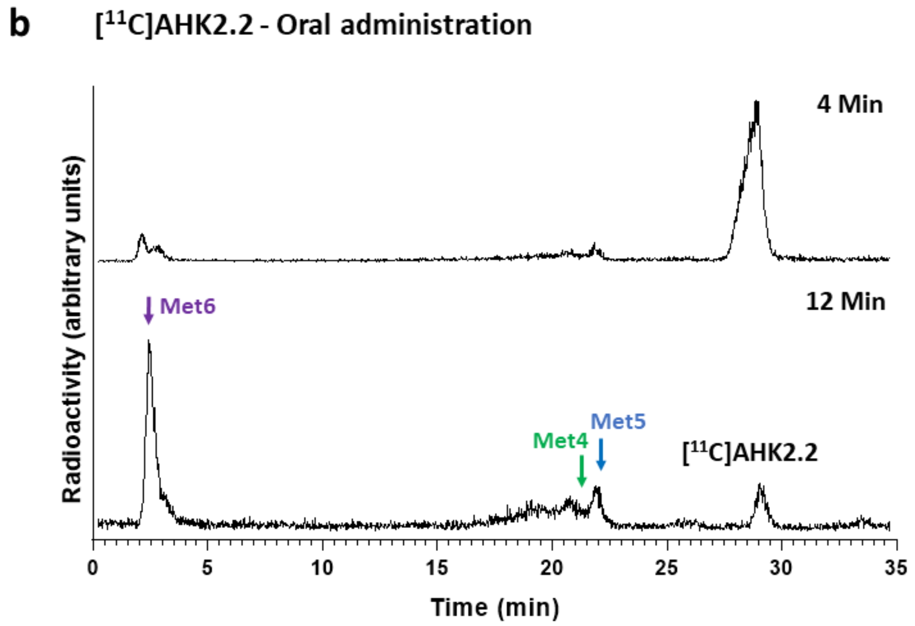

Figure S6. a) Representative arterial plasma Time Activity Curves (TACs) after oral administration of [^{11}C]AHK2.1 at [1 μg/Kg] (left) and [5 mg/Kg] (right); b) graphic representation of plasma concentration over time of AHK2 after intravenous administration of ^{11}C-labelled AHK2 at [1 μg/Kg] (left) and [5 mg/Kg] (right). The experimental values of unmetabolized parent compound (dots in the figure) were fitted using Hill type function (line) to obtain a continuous time course of plasma concentration of parent compound; c) metabolite-corrected arterial plasma TACs after intravenous administration of ^{11}C-labelled AHK2 at [1 μg/Kg] (left) and [5 mg/Kg] (right).

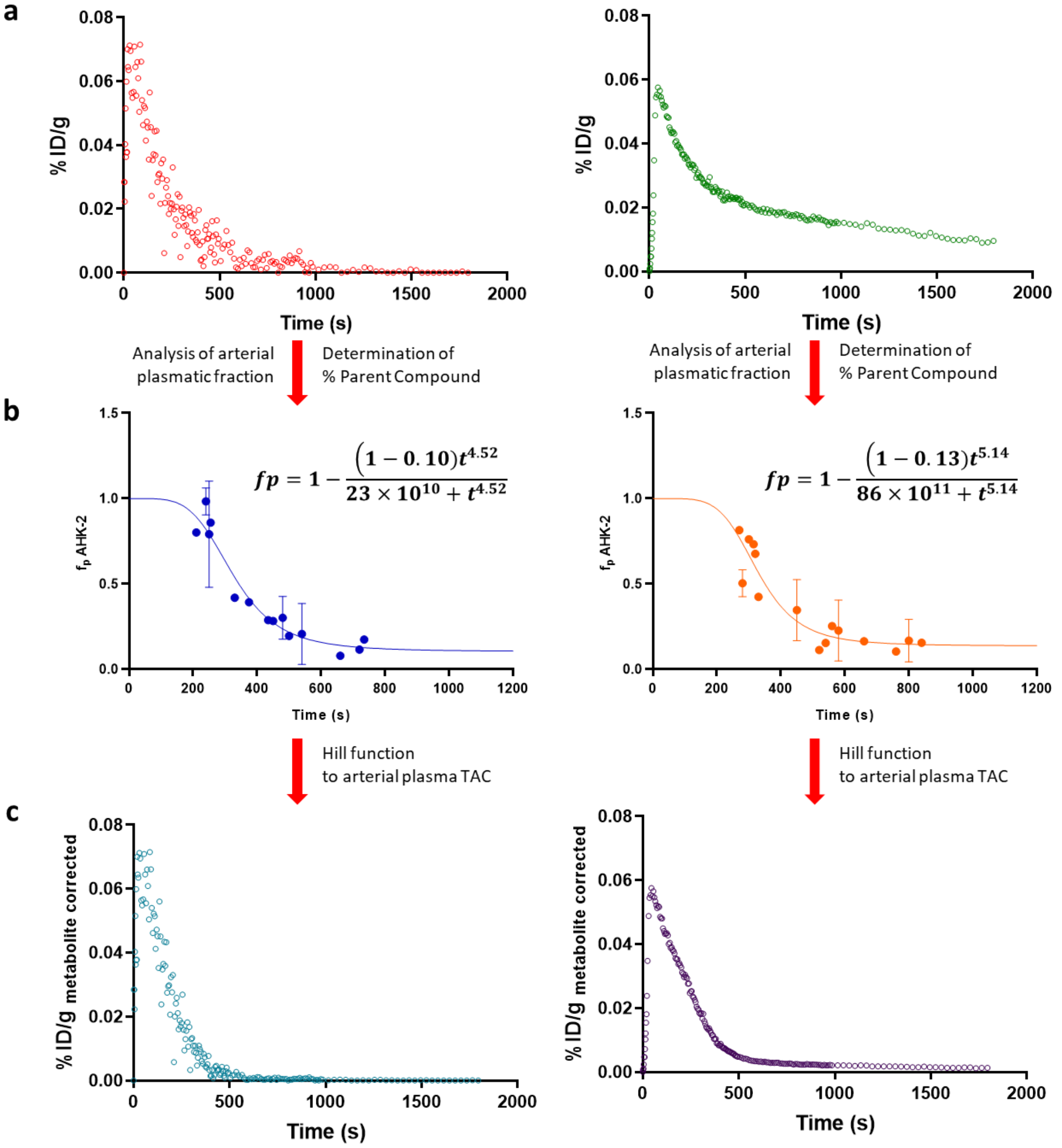

Figure S7. Biodistribution of [^{11}C]AHK2.1 (a) and [^{11}C]AHK2.2 (b) after oral administration (dose = 1 µg/Kg). Representative whole body coronal PET images (projections) obtained at different time points after administration. PET images have been co-registered with a representative CT slice for the anatomical localization of the radioactive signal. Scale bar in in Standard Uptake Value (SUV) units.

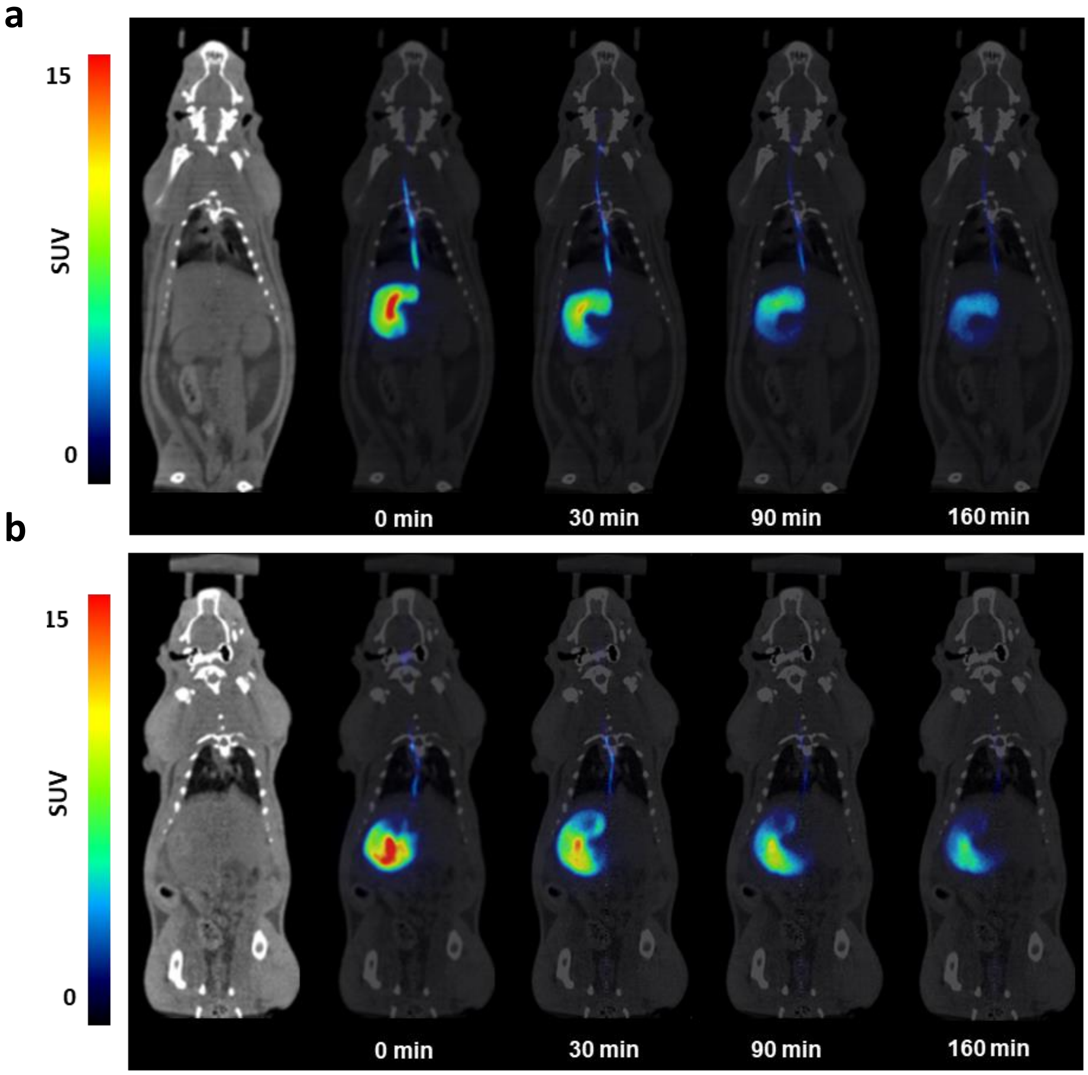

Figure S8. ^{1}H-NMR (400 MHz, CD_3OD) and ^{13}C-NMR (101 MHz, CD_3OD) spectra of compound AHK2 (HCl salt).

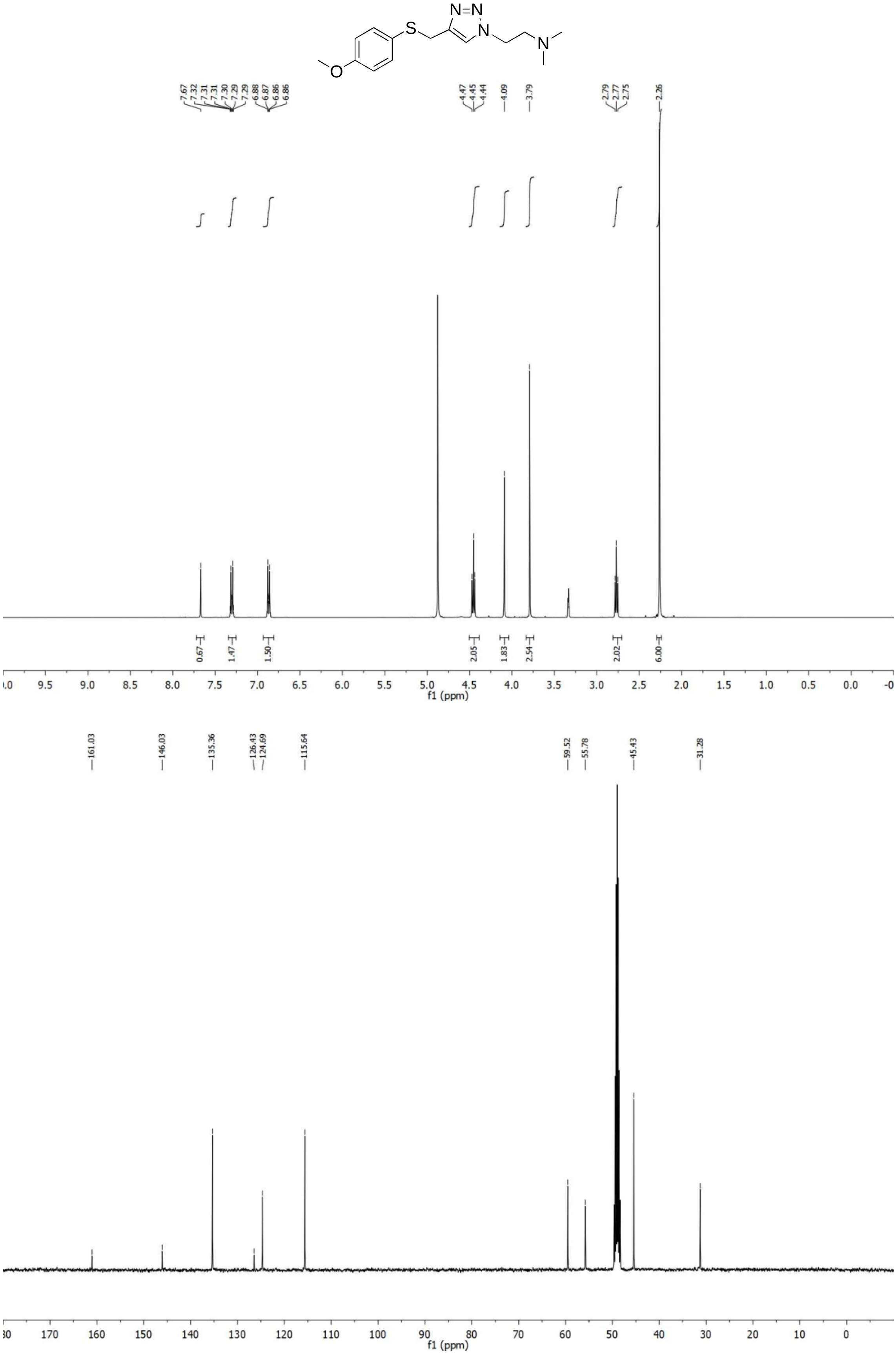

Figure S9. ^{1}H-NMR (400 MHz, CD_3OD) and ^{13}C-NMR (101 MHz, CD_3OD) spectra of Precursor **1** (HCl salt).

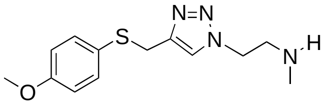

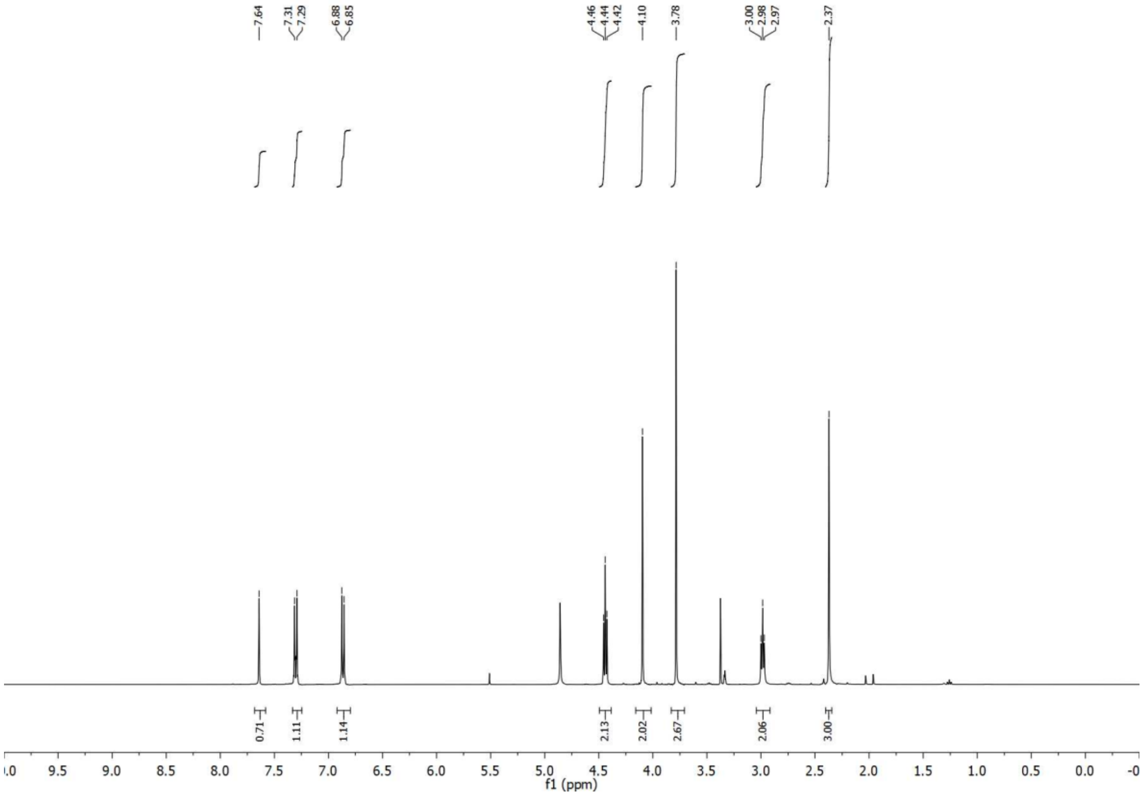

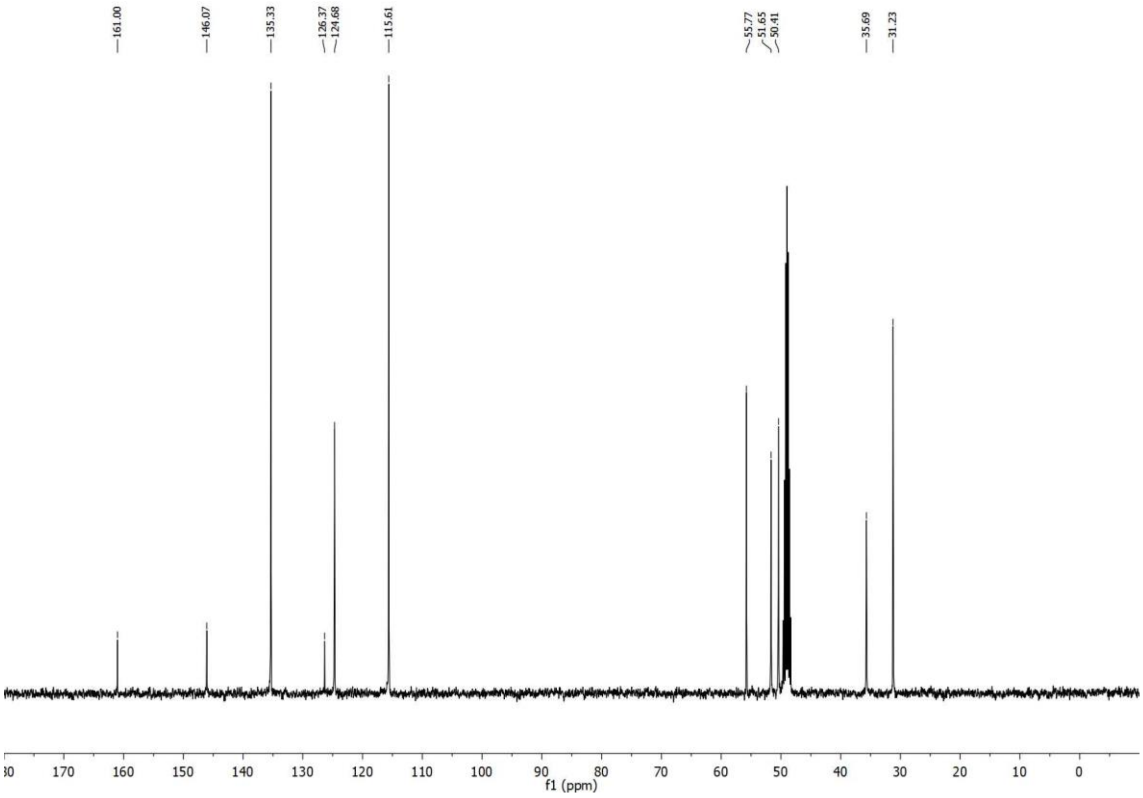

Figure S10. ^{1}H-NMR (400 MHz, CD_3OD, HCl salt) and ^{13}C-NMR (101 MHz, $CDCl_3$) spectra of Precursor **2**.

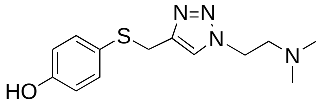

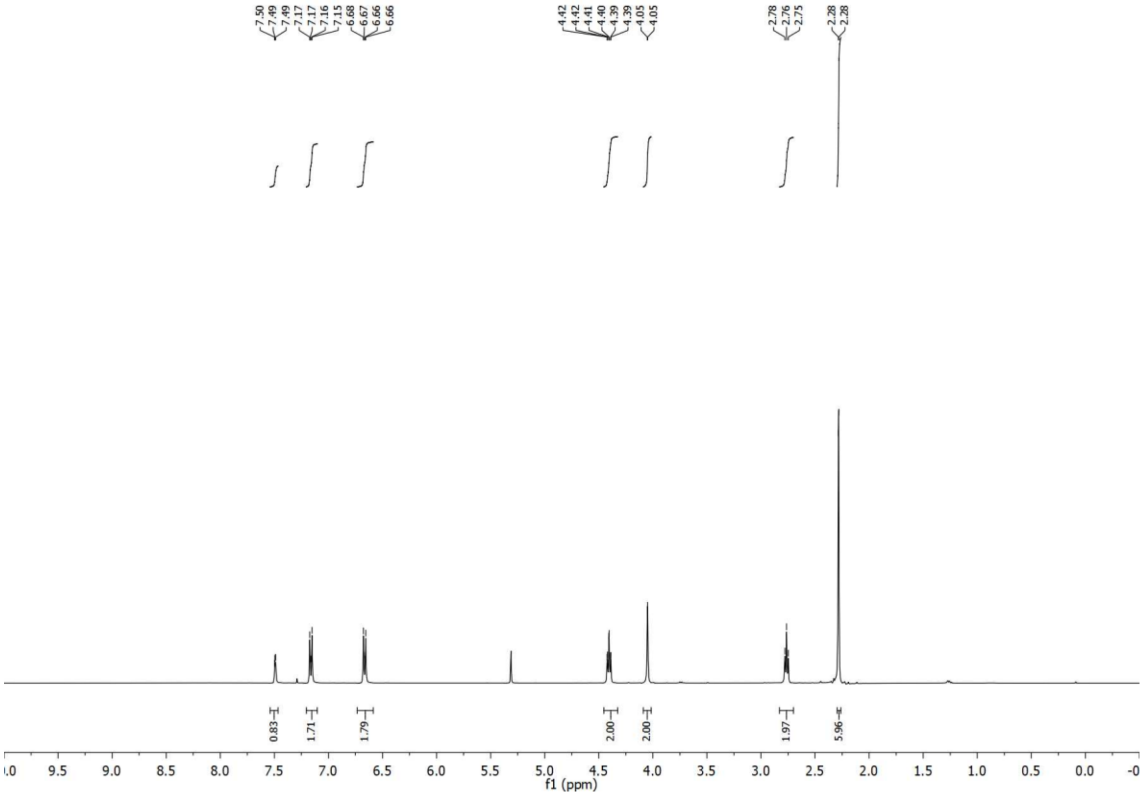

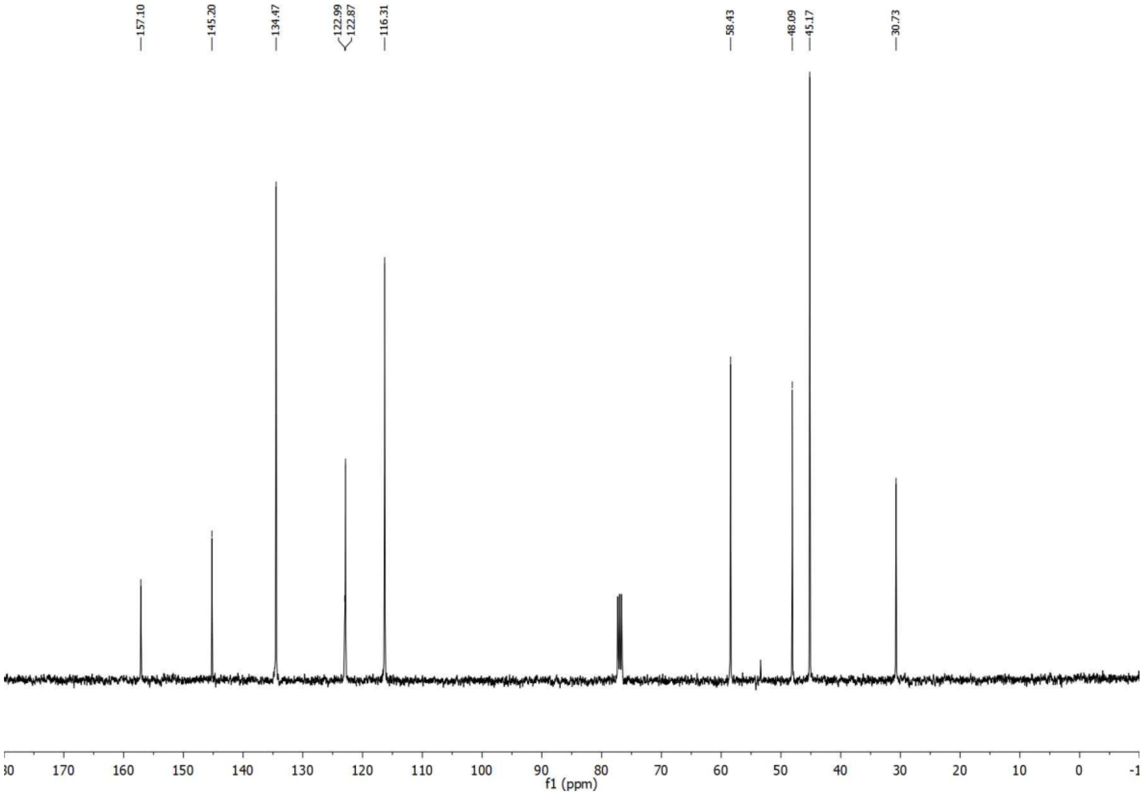

Figure S11. ^{1}H-NMR (400 MHz, CD_3OD) and ^{13}C-NMR (101 MHz, CD_3OD) spectra of Precursor **3** (HCl salt).

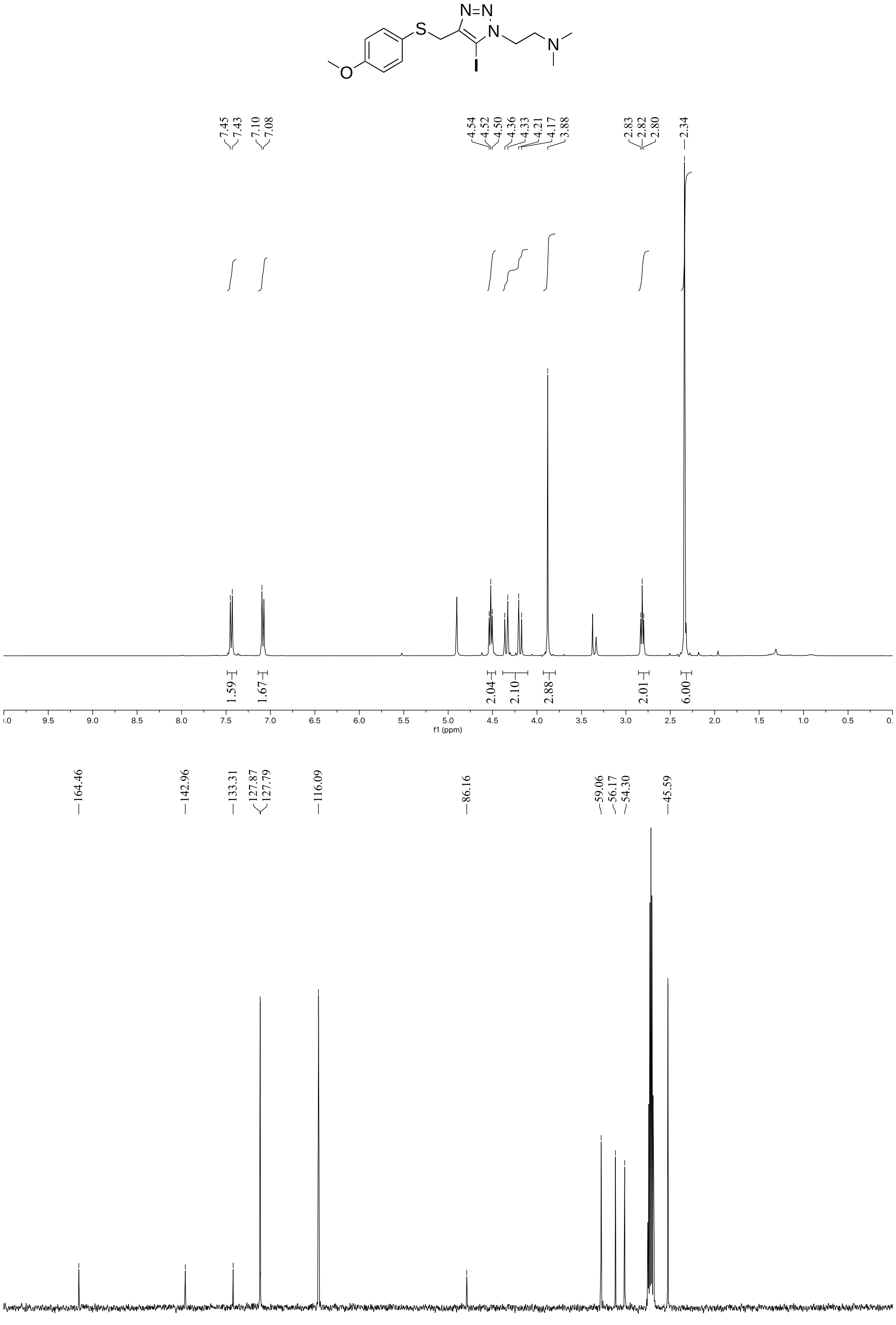

Figure S12. HPLC peaks and HR-MS spectra selected for m/z: 309.1 $[M+H]^+$, corresponding to mono-oxidized metabolites of AHK2 obtained in the in vitro metabolism assay. Sulfoxide Met 5 and an isobaric N-oxide are unambiguously detected.

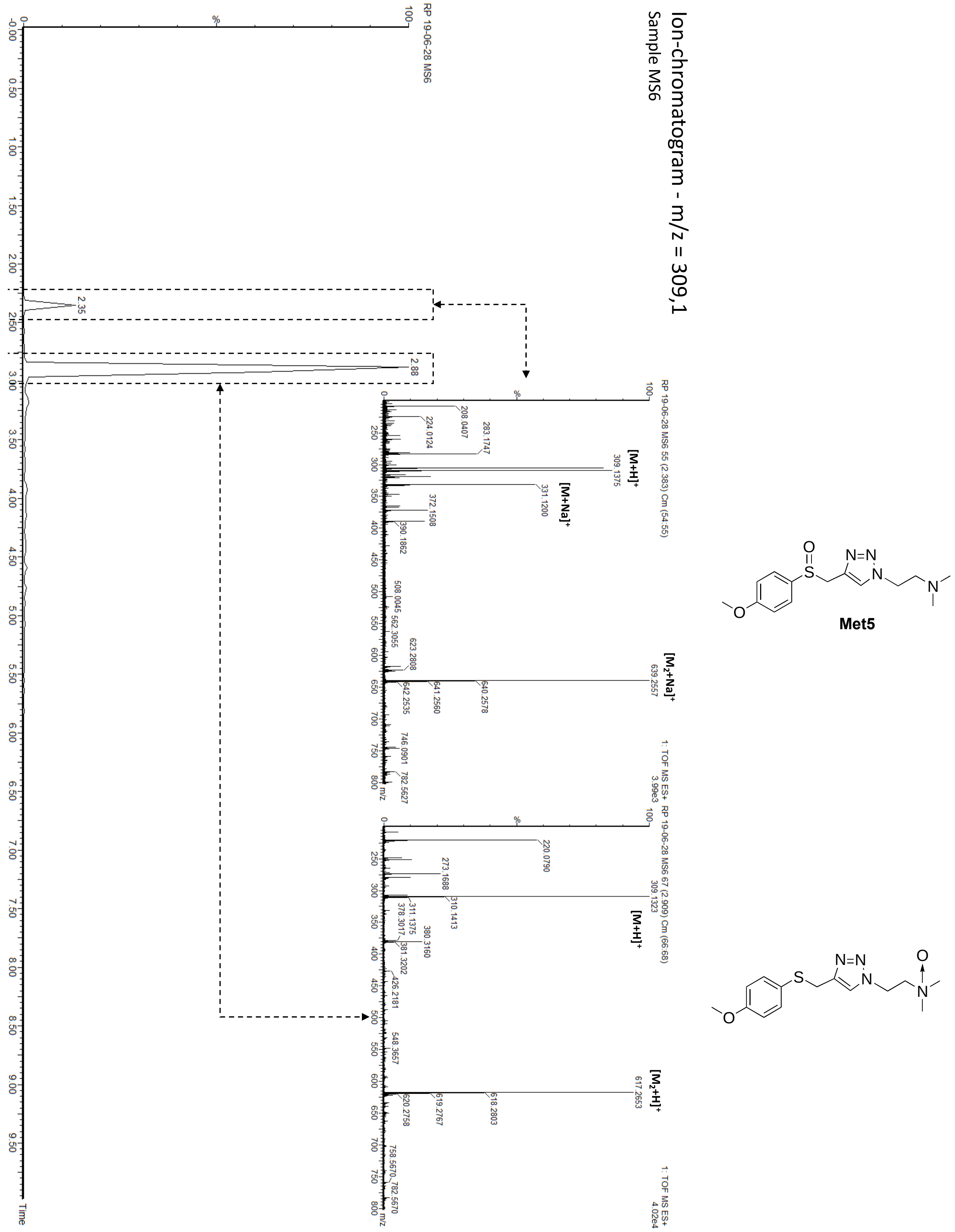

Supplement: Supplementary file 1 [file biomedicines-11-00253-s001.zip › biomedicines-2099911-supplementary.pdf]
